# Calcium-induced tetramerization and zinc chelation shield human calprotectin from degradation by host and bacterial extracellular proteases†
†Electronic supplementary information (ESI) available: Supplementary Tables S1–S6, supplementary Fig. S1–S22. Data for protease activity controls are included in Fig. S19–S22. See DOI: 10.1039/c5sc03287c


**DOI:** 10.1039/c5sc03287c

**Published:** 2015-11-23

**Authors:** Jules R. Stephan, Elizabeth M. Nolan

**Affiliations:** a Department of Chemistry , Massachusetts Institute of Technology , Cambridge , MA 02139 , USA . Email: lnolan@mit.edu ; Fax: +1-617-324-0505 ; Tel: +1-617-452-2495

## Abstract

Calprotectin (CP, S100A8/S100A9 oligomer, MRP-8/14 oligomer, calgranulins A and B) is a protein component of the innate immune system that contributes to the metal-withholding response by sequestering bioavailable transition metal ions at sites of infection. Human CP employs Ca(ii) ions to modulate its quaternary structure, transition metal binding properties, and antimicrobial activity. In this work, we report the discovery that Ca(ii)-induced self-association of human CP to afford heterotetramers protects the protein scaffold from degradation by host serine proteases. We present the design and characterization of two new human CP-Ser variants, S100A8(C42S)(I60E)/S100A9(C3S) and S100A8(C42S)(I60K)/S100A9(C3S), that exhibit defective tetramerization properties. Analytical size exclusion chromatography and analytical ultracentrifugation reveal that both variants, hereafter I60E and I60K, persist as heterodimers in the presence of Ca(ii) only, and form heterotetramers in the presence of Mn(ii) only and both Ca(ii) and Mn(ii). Coordination to Fe(ii) also causes I60E and I60K to form heterotetramers in both the absence and presence of Ca(ii). The Ca(ii)-bound I60E and I60K heterodimers are readily degraded by trypsin, chymotrypsin, and human neutrophil elastase, whereas the Ca(ii)-bound CP-Ser heterotetramers and the Ca(ii)- and Mn(ii)-bound I60E and I60K heterotetramers are resistant to degradation by these host proteases. The staphylococcal extracellular protease GluC cuts the S100A8 subunit of CP-Ser at the C-terminal end of residue 89 to afford a ΔSHKE variant. The GluC cleavage site is in close proximity to the His_3_Asp metal-binding site, which coordinates Zn(ii) with high affinity, and Zn(ii) chelation protects the S100A8 subunit from GluC cleavage. Taken together, these results provide new insight into how Ca(ii) ions and transition metals modulate the chemistry and biology of CP, and indicate that coordination to divalent cations transforms human CP into a protease-resistant form and enables innate immune function in the hostile conditions of an infection site.

## Abstract 2

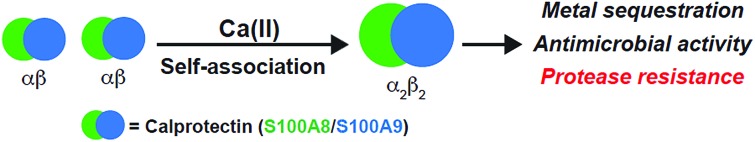
Coordination to divalent cations protects the human host-defense protein calprotectin from proteolytic degradation.

## Introduction

Calprotectin (CP, S100A8/S100A9 oligomer, MRP-8/14 oligomer, calgranulins A and B) is an antimicrobial protein and important player in the human innate immune response and inflammation.^1^–^7^ Neutrophils and epithelial cells express CP and release the protein into the extracellular space where it functions as an antimicrobial factor and a mediator of inflammation.^8^–^13^ Because of its remarkable biophysical properties and coordination chemistry, CP sequesters transition metal ions at sites of infection and thereby participates in the host metal-ion withholding response.^13^–^24^ In addition, CP is reported to be an endogenous ligand of toll-like receptor 4 (TLR4) and thereby contributes to the host inflammatory response.^11^,^25^ Despite these established roles, little is known about the fate of CP following its release into the extracellular space. In considering the extracellular functions and fate of CP, we questioned how CP copes with the harsh environments that it encounters at sites of infection, inflammation, and in the intestinal lumen. These locales harbor a number of factors that pose challenges for the host-defense machinery, which include proteases as well as reactive oxygen and nitrogen species. Protease resistance is a hallmark of host-defense peptides, such as the defensins,^26^,^27^ that are abundant in these environments, and we reasoned that CP must resist attack by extracellular proteases. Indeed, full-length murine S100A8 and S100A9 subunits have been detected in murine tissue abscesses infected with *Staphylococcus aureus*.^16^ We hypothesized that changes in the quaternary structure of CP resulting from metal chelation contribute to protease resistance as described below. This notion is inspired by the defensins, which utilize disulfide bonds to achieve a three-stranded β-sheet fold that stabilizes the peptide backbone from proteases,^28^,^29^ and metal-chelating proteins such as lactoferrin, which exhibits enhanced resistance to degradation by trypsin and chymotrypsin in its Fe(iii)-bound form.^30^,^31^

Human CP exhibits complex oligomerization^32^–^37^ and metal-binding properties.^19^–^24^ It is a hetero-oligomer of the Ca(ii)-binding S100 proteins S100A8 (93 amino acids, 10.8 kDa, α subunit) and S100A9 (114 amino acids, 13.2 kDa, β subunit).^32^,^34^,^35^ Apo human CP exists as a heterodimer (αβ).^32^ Each CP subunit exhibits two EF-hand domains, and Ca(ii) ion binding results in formation of the CP heterotetramer (α_2_β_2_).^34^–^37^ Each heterodimer also harbors two sites that form at the S100A8/S100A9 interface for chelating transition metal ions.^17^,^19^–^24^,^36^ In addition to affecting quaternary structure, Ca(ii) ions also modulate the transition metal binding properties and antimicrobial activity of CP.^19^–^22^,^24^ Because extracellular Ca(ii) levels are high, the CP heterotetramer is expected to be a relevant and abundant extracellular form.^19^,^38^ We therefore questioned whether Ca(ii) binding to CP and consequent formation of the heterotetramer protects the scaffold against degradation by host and bacterial proteases. The proteolytic stability of CP is largely unexplored, and some of the results from published studies appear to be conflicting. Two independent studies demonstrated that the CP heterooligomer is more protease resistant than the S100A8 and S100A9 homodimers.^39^,^40^ One of these reports also found that CP in human leukocyte cell lysate was a poor substrate for trypsin and proteinase K in both the absence and presence of a Ca(ii) and Zn(ii) supplement.^39^ A recent investigation evaluated the susceptibility of CP collected from human fecal matter to trypsin hydrolysis, and concluded that CP in fecal samples is susceptible to trypsin degradation.^41^ A complicating factor in evaluating and comparing the outcomes of these studies is that the speciation of the CP substrate is unknown and likely multifaceted (*i.e.* oligomeric state, metal-free *versus* metal-bound). To clarify whether CP resists protease attack and test our hypothesis that speciation plays a role, we sought to systematically evaluate how metal ions and quaternary structure influence proteolytic stability.

In this work, we report that Ca(ii)-induced tetramerization protects CP from host serine proteases. Moreover, we establish that Zn(ii) complexation protects the S100A8 C-terminus from the staphylococcal serine protease GluC. These data support a new dimension to how Ca(ii) ions and transition metals modulate the function and fate of extracellular CP, indicating that Ca(ii) binding allows CP to sequester transition metals and resist attack by host proteases.

## Results and discussion

### Design and preparation of tetramer-deficient variants of calprotectin

**Fig. 1:**
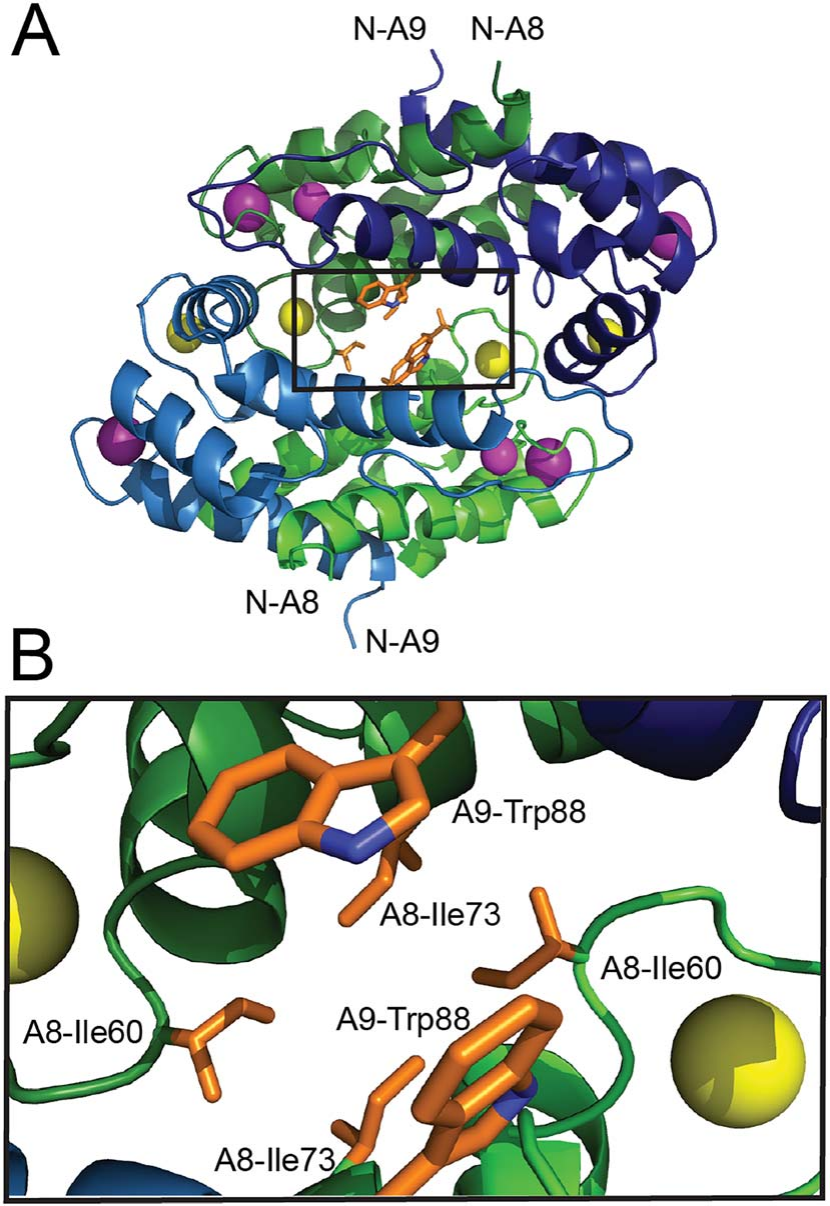
Crystal structure of the Mn(ii)-, Ca(ii)-, and Na(i)-bound human CP-Ser heterotetramer (PDB: ; 4XJK).^22^ (A) The heterotetramer with select hydrophobic residues at the tetramer interface shown as sticks. The outlined box indicates the region displayed in panel B. (B) A zoomed-in view of the heterotetramer interface that illustrates the clustering of hydrophobic side chains. The green chains are the S100A8 subunits and the blue chains are the S100A9 subunits. One heterodimer is depicted in dark shading and the second heterodimer is depicted in light shading. The Ca(ii) ions are shown as yellow spheres, the Na(i) ions are shown as purple spheres, and the Mn(ii) ions are shown as magenta spheres. The amino acid sequences for the S100A8 and S100A9 subunits are provided in Fig. S1, ESI.†

In order to investigate whether Ca(ii)-induced tetramerization of human CP affords protease resistance, we sought to compare the stabilities of Ca(ii)-bound heterodimers and Ca(ii)-bound heterotetramers in protease degradation assays. These experiments require CP variants that remain heterodimeric following Ca(ii) complexation. The crystal structure of Mn(ii)-, Ca(ii)-, and Na(i)-bound CP-Ser (PDB ; 4XJK 22) reveals that the tetramer interface between CP heterodimers is ≈3700 Å^2^ and largely comprised of contacts between the S100A8 subunits. Moreover, a cluster of hydrophobic residues occurs at the heterotetramer interface (Fig. 1). The S100A8 subunit of each heterodimer contributes (A8)Ile60, (A8)Ile73, and (A8)Ile76 (not shown), and the S100A9 subunits contribute (A9)Trp88. We hypothesized that this hydrophobic region is a “hot spot” for tetramerization,^42^ and reasoned that point mutations of the hydrophobic residues may provide the requisite CP-Ser variants that retain the ability to coordinate Ca(ii) but cannot undergo Ca(ii)-induced tetramerization. We mutated (A8)Ile60 to Glu and Lys as a case study, and focus on these variants herein. We reasoned that introduction of charged residues at position 60 of S100A8 should disfavor tetramerization by decreasing the hydrophobic driving force and introducing electrostatic repulsion. Moreover, the parity of charges introduced by the Glu and Lys mutations provides a control to minimize the likelihood that any biophysical or functional differences between CP and the variants are artifacts of a given amino acid substitution. Lastly, it was important to preserve the metal-binding properties of CP. Because (A8)Ile60 does not contribute to Ca(ii)-ion coordination and is distant from both the transition metal binding sites (His_3_Asp, His_4/6_), we reasoned that mutating this residue would result in negligible perturbation to the metal-ion coordination spheres. We abbreviate the CP-Ser variants bearing the (A8)Ile60Glu and (A8)Ile60Lys mutations I60E and I60K, respectively.

We prepared pET41a-S100A8(C42S)(I60E) and pET41a-S100A8(C42S)(I60K) plasmids by site-directed mutagenesis. We overexpressed and purified I60E and I60K as described previously for CP-Ser.^19^ The variants were obtained in yields of approximately 28 and 15 mg L^–1^ of culture, respectively. The proteins were isolated as the αβ heterodimers in high purity, and the S100A8 and S100A9 subunits were present in equal amounts as judged by gel electrophoresis (Fig. S2, ESI†). The identities of the subunits were confirmed by mass spectrometry (Table S3†), and circular dichroism spectroscopy verified that the mutations did not affect the overall α-helical secondary structure of the CP scaffold (Fig. S3†). We employed two established competition assays to probe the metal-binding properties of I60E and I60K.^20^ First, a competition titration employing the fluorescent metal-ion sensor Zinpyr-1 (ZP1, apparent *K*_d1,Mn_ = 550 nM)^43^ confirmed that I60E and I60K coordinate Mn(ii) with high affinity when excess Ca(ii) is included in the buffer. Like CP-Ser, both variants outcompeted ZP1 for Mn(ii) in the presence of excess Ca(ii), which indicated that the apparent *K*_d,Mn_ value of each variant is less than 550 nM (Fig. S4†). Moreover, titration of Ca(ii) into a solution containing ZP1, Mn(ii), and protein demonstrated that CP-Ser, I60E, and I60K show comparable Ca(ii)-dependent Mn(ii)-binding properties. Each protein required ≈20 equivalents of Ca(ii) to fully sequester Mn(ii) from ZP1 (Fig. S5†). Taken together, the results from these titrations demonstrate that the Mn(ii)-binding properties of I60E and I60K are Ca(ii)-dependent, and that these variants behave like CP-Ser, at least in the context of ZP1 competition assays.

### I60E and I60K mutations disrupt Ca(ii)-induced tetramerization

**Fig. 2:**
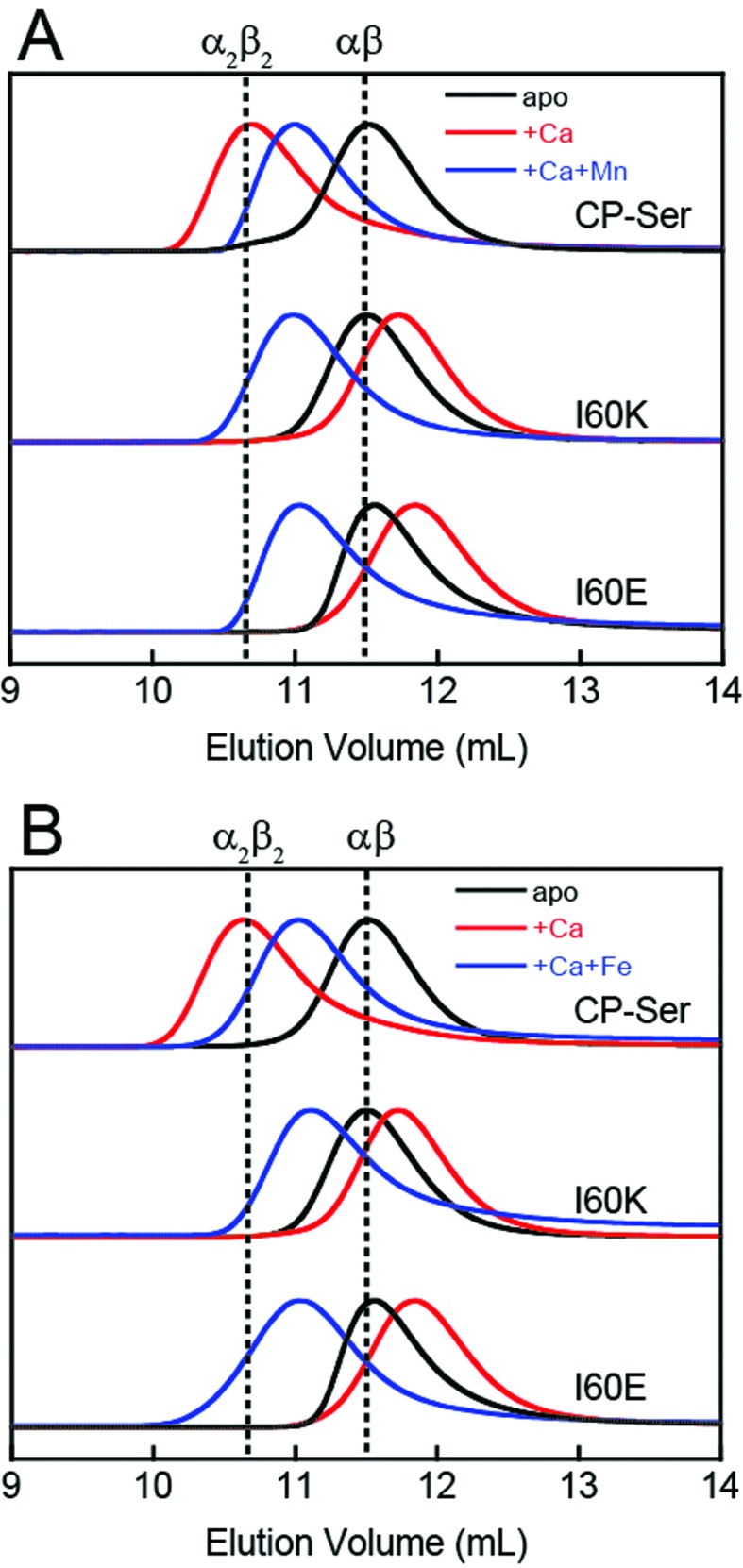
Analytical SEC traces of CP-Ser, I60E and I60K (30 μM) in absence and presence of various metals (75 mM HEPES, 100 mM NaCl, pH 7.5, 4 °C). (A) Black traces, no metal added. Red traces, 600 μM Ca(ii) in running buffer and protein sample. Blue traces, 600 μM Ca(ii) in running buffer and sample, 33 μM Mn(ii) in the protein sample only. (B) Black traces, no metal added. Red traces, 600 μM Ca(ii) in running buffer and protein sample. Blue traces, 600 μM Ca(ii) in running buffer and sample, 33 μM Fe(ii) in the protein sample only. Each chromatogram was normalized to a maximum peak value of 1. The vertical dashed lines indicate the peak elution volumes of the αβ (–Ca) and the α_2_β_2_ (+Ca) forms of CP-Ser. Chromatograms for CP-Ser, I60E and I60K pre-incubated with Mn(ii) only and Fe(ii) only are provided in Fig. S6 and S7.†

To probe whether the I60E and I60K variants display perturbed oligomerization properties in the presence of Ca(ii), we employed analytical size-exclusion chromatography (SEC) and determined the elution volumes and corresponding molecular weights of the proteins in the absence and presence of excess Ca(ii) (Fig. 2 and Table S4†). In this set of experiments, apo CP-Ser (30 μM) exhibited a peak elution volume at 11.5 mL (≈35 kDa), and the peak shifted to 10.7 mL (≈48 kDa) when excess Ca(ii) (600 μM) was included in the running buffer (75 mM HEPES, 100 mM NaCl, pH 7.5). This behavior was in agreement with our prior analytical SEC studies of CP-Ser,^19^ and demonstrates the expected Ca(ii)-dependent formation of the α_2_β_2_ tetramer (Fig. 2). Apo I60E and I60K exhibited the same peak elution volume as apo CP-Ser, indicating that the mutants are heterodimers in the absence of Ca(ii) (Fig. 2). In contrast to CP-Ser, both I60E and I60K exhibited a slight peak shift to a later elution volume (11.8 mL, ≈31 kDa) when Ca(ii) (600 μM) was included in the running buffer (Fig. 2). This behavior indicates that (i) I60E and I60K bind Ca(ii), which is in agreement with the competition titrations described above; (ii) Ca(ii) complexation causes the hydrodynamic radii of I60E and I60K to decrease relative to the apo proteins; and (iii) Ca(ii)-bound I60E and I60K do not form α_2_β_2_ heterotetramers under these experimental conditions. To probe whether the protein concentration influences the oligomerization properties, we increased the I60K and Ca(ii) concentrations to 500 μM and 10 mM, respectively, and observed no evidence for heterotetramer formation (Fig. S6†).

To confirm that the I60E and I60K mutations disrupted Ca(ii)-induced tetramerization, we employed analytical ultracentrifugation (AUC) and determined sedimentation coefficients for CP-Ser, I60E, and I60K in the absence and presence of 20 equivalents of Ca(ii) (75 mM HEPES, 100 mM NaCl, pH 7.5). We obtained theoretical sedimentation coefficients (*s*_20,w_) using HYDROPRO^44^ and the available CP-Ser crystal structures as models (PDB ; 1XK4,^36^; 4GGF,^23^ and ; 4XJK 22). HYDROPRO afforded predicted sedimentation coefficients of 2.1 S and 3.7 S for the heterodimer and heterotetramer, respectively. We also determined the maximum theoretical sedimentation coefficients for the heterodimer and heterotetramer to be 2.8 S and 4.5 S, respectively.^45^

**Fig. 3:**
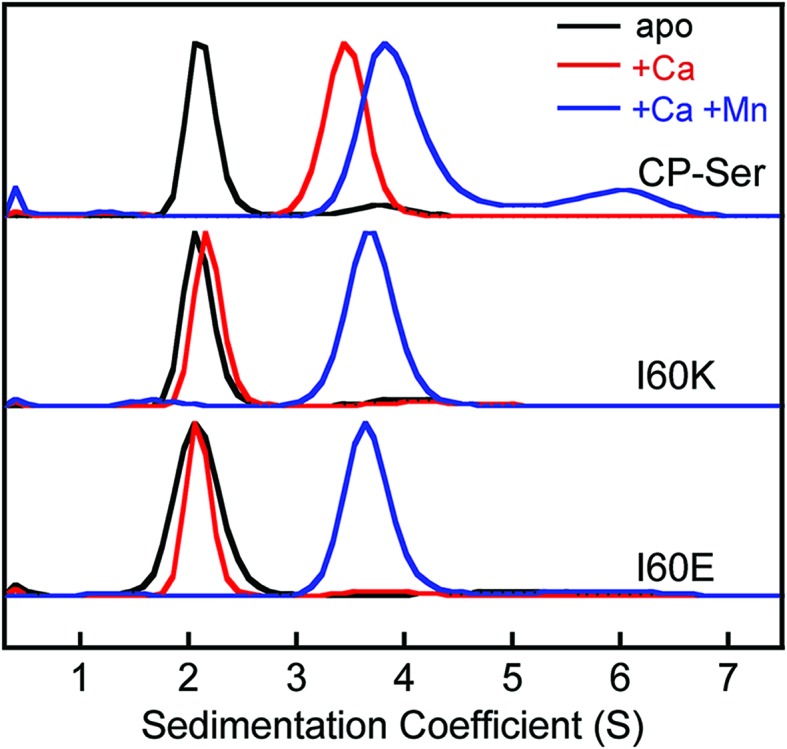
Sedimentation distributions of CP-Ser, I60E, and I60K (27.5 μM) obtained with the *c*(*s*) model in SEDFIT (75 mM HEPES, 100 mM NaCl, pH 7.5, 20 °C). Black traces, no metal added. The CP-Ser sample included 550 μM EDTA. Red traces, 550 μM of Ca(ii) in the sample. Blue traces, 550 μM Ca(ii) and 27.5 μM Mn(ii) in the sample. Each sedimentation distribution is normalized to the maximum *c*(*s*).

The sedimentation distributions of apo CP-Ser, I60E, and I60K determined using SEDFIT each displayed a single major species with an S value between 2.3 S and 2.4 S (Fig. 3 and S8†), which are in agreement with the predicted S value for the heterodimer. When 20 equivalents of Ca(ii) were added to the sample, the sedimentation distribution for CP-Ser was dominated by a single species at 3.7 S, which corresponds to the predicted S value for the α_2_β_2_ heterotetramer (Fig. 3). In contrast, the major species for both I60E and I60K remained at ≈2.3 S when the samples contained excess Ca(ii). In agreement with the SEC experiments, the AUC data demonstrate that I60E and I60K remain heterodimeric with Ca(ii). Analysis of the AUC data using DCDT+ afforded the same conclusions (Fig. S9†).

### I60E and I60K variants form heterotetramers in the presence of Mn(ii) or Fe(ii)

CP coordinates Mn(ii) and Fe(ii) with high affinity at a hexahistidine site comprised of (A8)His17, (A8)His27, (A9)His91, (A9)His95, (A9)His103, and (A9)His105.^21^–^24^ The Mn(ii) and Fe(ii) affinities at this site depend on the presence of Ca(ii) ions, and stoichiometric binding of Mn(ii) and Fe(ii) is observed at the His_6_ site when excess Ca(ii) is present.^20^,^24^ Moreover, addition of Mn(ii) or Fe(ii) to CP-Ser results in conformational change.^20^,^21^,^24^ For instance, prior analytical SEC studies revealed that preincubation of CP-Ser (αβ, –Ca) with 10 equivalents of Mn(ii) results in a new peak (11.0 mL, ≈43 kDa) that elutes between the apo heterodimer and Ca(ii)-bound heterotetramer (Fig. S6†).^20^,^21^ In contrast, pre-incubation of I60E and I60K with 10 equivalents of Mn(ii) and no Ca(ii) afforded peaks with the same peak elution volumes as the apo heterodimer (11.5 mL, ≈35 kDa). Nevertheless, the peaks markedly broadened under these conditions, indicating that the samples contain a mixture of heterodimers as well as heterotetramers (Fig. S6†). To determine the effect of Mn(ii) and Ca(ii) on the elution properties of I60K and I60E, the proteins (30 μM) were pre-incubated with 20 equivalents of Ca(ii) and 1.1 equivalents of Mn(ii), and excess Ca(ii) (600 μM) was included in the running buffer. Under these conditions, I60E and I60K afforded peaks with elution volumes of ≈11 mL (Fig. 2). These chromatograms suggest the combination of Mn(ii) and excess Ca(ii) causes I60E and I60K to completely form heterotetramers.

We employed AUC to further investigate how Mn(ii) coordination influences the quaternary structure of CP-Ser, I60E, and I60K in the absence or presence of Ca(ii). The sedimentation distributions for samples containing CP-Ser and one equivalent of Mn(ii) reveal two main species at 2.4 S and 4.1 S (Fig. S8†). The species at 4.1 S comprises ≈65% of the distribution area. In the absence of Ca(ii), CP-Ser coordinates Mn(ii) with relatively low affinity and the reported dissociation constant at the His_6_ site is in the micromolar range (*K*_d1_ ≈ 5 μM by room-temperature electron paramagnetic resonance spectroscopy).^20^,^22^ Using this *K*_d_ value and the CP-Ser and Mn(ii) concentrations employed for the AUC sample, we calculate that the sample contained ≈34% apo and ≈66% Mn(ii)-bound CP-Ser. On the basis of this analysis, we assign the 2.4 S species to the apo CP-Ser heterodimer and the 4.1 S species to a Mn(ii)-bound heterotetramer. This analysis informs our prior SEC results that revealed addition of Mn(ii) to CP-Ser causes a shift in peak elution volume (*vide supra*), and we conclude that apo heterodimers and Mn(ii)-bound heterotetramers are present in the sample. Moreover, when I60E and I60K were pre-incubated with one equivalent of Mn(ii), the sedimentation distributions exhibited the same dimer (≈2.3 S) and tetramer species (≈4.0 S) (Fig. S8†); however, the dimer comprised ≈80% of the total area, indicating that Mn(ii)-induced tetramerization is disrupted in the I60E and I60K variants. This analysis is consistent with the SEC results, which showed a peak elution volume consistent with heterodimers and marked peak broadening indicative of heterotetramers.

When 20 equivalents of Ca(ii) and one equivalent of Mn(ii) were added to the buffer, CP-Ser, I60E, and I60K exhibited similar sedimentation distributions (Fig. 3). The major species appeared at ≈4.0 S for all three proteins, which we assign to the Ca(ii)- and Mn(ii)-bound heterotetramer. The CP-Ser distribution also contained a less abundant species at 6.5 S. The theoretical maximum sedimentation coefficient for the CP tetramer is ≈4.5 S, suggesting that a higher order oligomer forms under these conditions. This species warrants further investigation, and is reminiscent of prior SEC studies that revealed low-abundance peaks with lower elution volumes (higher molecular weight) than the α_2_β_2_ heterotetramer.^19^ In total, the AUC results are in agreement with those obtained from SEC and indicate that (i) the quaternary structure of CP is modulated by both Ca(ii) and Mn(ii), (ii) heterotetramerization is not restricted to Ca(ii) because the CP-Ser samples containing only Mn(ii) also form heterotetramers, and (iii) the I60E and I60K mutations do not preclude formation of tetramers when Ca(ii) and Mn(ii) are present. Moreover, crystal structures of Ca(ii)- and Mn(ii)-bound CP-Ser reveal heterotetramers,^22^,^23^ and the current work confirms that heterotetramers also form in solution.

We also employed SEC to examine the effect of Fe(ii) on the oligomerization properties of CP-Ser, I60E, and I60K in the absence and presence of excess Ca(ii). In agreement with our previous work, we observed a shift in peak elution volume following addition of Fe(ii) to CP-Ser.^24^ After preincubation with 10 equivalents of Fe(ii), CP-Ser, I60E, and I60K (30 μM) shifted to an earlier peak elution volume (11.1 mL, ≈43 kDa) (Fig. S7†). In contrast to the Mn(ii)-only I60E and I60K samples (Fig. S6†), the chromatograms for the Fe(ii)-only samples exhibited a complete shift to earlier elution volumes and no broadening, which indicated that addition of Fe(ii) favored tetramerization more than Mn(ii) alone. Moreover, preincubation of the proteins with 1.1 equivalents of Fe(ii) and 20 equivalents of Ca(ii) afforded the same peak elution volume of 11.1 mL (Fig. 2B). The higher affinity of CP for Fe(ii) than Mn(ii) at the His_6_ site may explain why Fe(ii) causes more tetramerization of I60E and I60K than Mn(ii) in the absence of Ca(ii).^24^ We note that one prior study reported Zn(ii)-induced formation of heterotetramers by mass spectrometry, albeit the Zn(ii)/CP stoichiometries obtained from this analysis are unlikely to be relevant because the number of bound Zn(ii) ions reported exceeds the number of Zn(ii)-binding sites per CP heterotetramer.^37^ Taken together, these studies illuminate the multifaceted and metal-dependent oligomerization properties of CP. Our data indicate that the I60 mutations abrogate Ca(ii)-induced tetramerization, but allow tetramerization to occur *via* transition metal binding. Nevertheless, the molecular basis for the metal-dependent oligomerization properties of CP-Ser and the I60 variants is unclear from standpoints of Ca(ii) coordination at the EF-hand domains as well as transition metal binding at the His_6_ site. Further characterization of these metal-dependent self-assembly pathways is an avenue for future work.

### Tetramerization protects calprotectin against host proteases

To ascertain whether Ca(ii)-induced tetramerization of CP-Ser confers resistance to proteolysis, we evaluated trypsin, chymotrypsin, and human neutrophil elastase (HNE) as host serine proteases in degradation assays. We selected trypsin, chymotrypsin, and HNE because we reasoned that co-localization of these proteases with CP is likely to occur *in vivo*. CP is expressed by the intestinal epithelium and serves as a biomarker for inflammation in the gut, where trypsin and chymotrypsin are abundant.^46^ Neutrophils are a major source of extracellular CP and also release HNE, the latter of which is stored in azurophilic granules.^9^,^47^,^48^ We employed Ca(ii)-bound CP-Ser, I60E, and I60K in these studies to compare the proteolytic stabilities of the Ca(ii)-bound heterotetramer (CP-Ser) and heterodimers (I60E and I60K). Because proteases such as trypsin and chymotrypsin are activated/stabilized by Ca(ii) ions, a degradation study of the apo CP-Ser heterodimer degradation would necessitate utilizing incompletely active proteases and direct comparison with the Ca(ii)-bound heterotetramer would not be possible. With the tetramer-deficient variants, it is possible to compare the stability of the Ca(ii)-bound dimer and the Ca(ii)-bound tetramer under the same experimental conditions that provide equally active proteases.

**Fig. 4:**
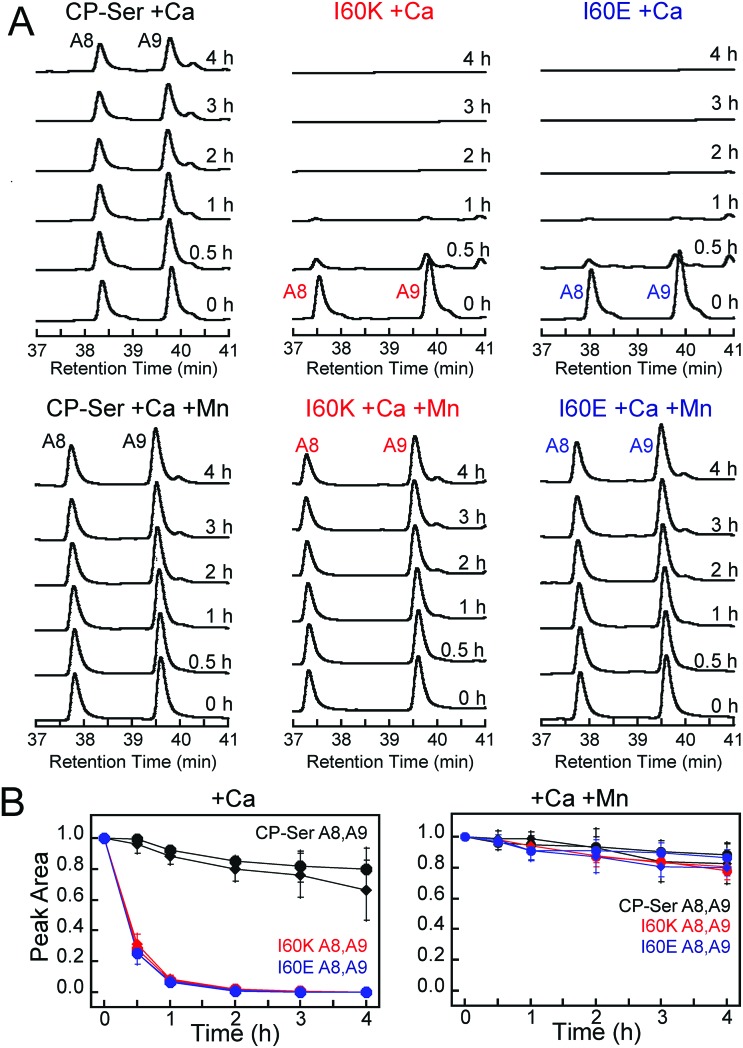
Susceptibility of Ca(ii)-bound CP-Ser, I60E, and I60K (30 μM) to degradation by trypsin (0.45 μM). (A) Representative HPLC traces illustrating the full-length S100A8 and S100A9 subunits following incubation with trypsin for 0–4 h at 37 °C (75 mM HEPES, 100 mM NaCl, ±1.5 mM CaCl_2_, ±30 μM MnCl_2_, pH 7.5). (B) Reduction in the S100A8 and S100A9 integrated peak areas (normalized to initial peak areas) as a function of time (mean ± SDM, *n* = 3). Full chromatograms are given in Fig. S11.†

**Fig. 5:**
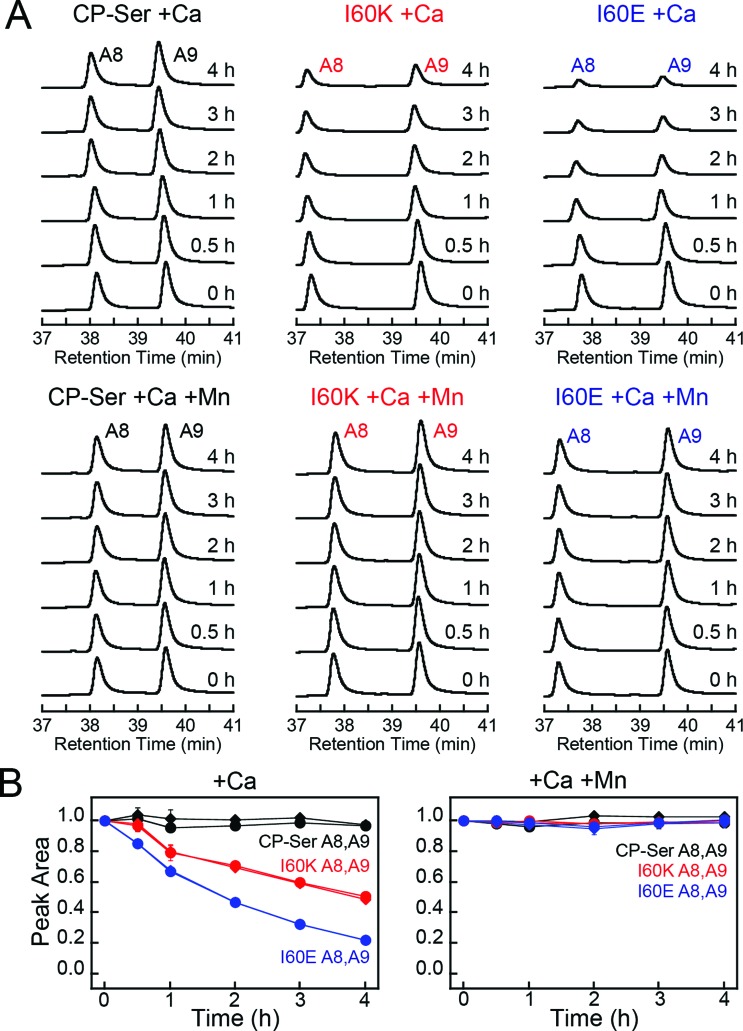
Susceptibility of Ca(ii)-bound CP-Ser, I60E, and I60K (30 μM) to degradation by HNE (0.3 μM). (A) Representative HPLC traces illustrating the full-length S100A8 and S100A9 subunits following incubation with HNE for 0–4 h at 37 °C (75 mM HEPES, 100 mM NaCl, ±1.5 mM CaCl_2_, ±30 μM MnCl_2_, pH 7.5). (B) Reduction in the S100A8 and S100A9 integrated peak areas (normalized to initial peak areas) as a function of time (mean ± SDM, *n* = 3). Full chromatograms are given in Fig. S13.†

We established an analytical HPLC protocol that affords separation of the S100A8 (≈38 min) and S100A9 (39.8 min) subunits on a C4 column. We confirmed the identity of each peak by LCMS (Fig. S10†), and used HPLC to monitor the proteolytic stability of each subunit over time. The chromatograms from the trypsin digestions revealed that S100A8 and S100A9 subunits of Ca(ii)-bound CP-Ser display resistance to trypsin whereas both subunits were rapidly degraded for Ca(ii)-bound I60E and I60K (Fig. 4A and S11†). Quantification of the S100A8 and S100A9 peak areas revealed that ≈80% of each CP-Ser subunit persisted after 4 h whereas both subunits were undetectable for I60K and I60E at this time point (Fig. 4B). Similar results were obtained with chymotrypsin (Fig. S12†) and HNE (Fig. 5 and S13†); both proteases degraded Ca(ii)-bound I60K and I60E more readily than Ca(ii)-bound CP-Ser. We also observed that HNE digested I60E more rapidly than I60K. The reason for the difference is unclear because HNE preferably cleaves after small hydrophobic residues, so the I60E and I60K mutations are not expected to affect the HNE cleavage pattern.^49^,^50^ Addition of both Mn(ii) and Ca(ii) to I60E and I60K, which causes heterotetramer formation (*vide supra*), resulted in enhanced stability to digestion by trypsin, chymotrypsin, and HNE. The corresponding chromatograms indicate negligible loss of full-length S100A8 and S100A9, and new species were not observed (Fig. 4, 5, and S11–S13†). Taken together, these assays demonstrate that tetramerization of the CP-Ser scaffold affords enhanced resistance to degradation by three host proteases.

### Proteolysis by trypsin abolishes antibacterial activity

The current model states that CP inhibits microbial growth in a Ca(ii)-dependent manner by sequestering essential transition metal ions in the extracellular space.^51^ This antibacterial mechanism requires that the Ca(ii) and transition metal binding sites at the S100A8/S100A9 interface remain intact and that the coordinated transition metals remain bound and inaccessible to an invading microbe. In accord with this model, it is reasonable to expect that proteolytic destruction of the metal-binding sites and heterooligomeric assembly results in attenuated antimicrobial activity.

We performed standard *in vitro* antimicrobial activity assays with I60E and I60K, and observed that these variants inhibited the growth of *E. coli* ATCC 29522 in a concentration-dependent manner that is comparable to CP-Ser (Fig. S14†). This result indicates that the defective self-assembly properties of I60E and I60K do not compromise *in vitro* antibacterial activity, at least against this *E. coli* strain. We note that I60E and I60K retain a functional His_6_ site, and prior studies established that the His_6_ site of CP contributes more to the *in vitro* antibacterial activity against *E. coli* than the His_3_Asp site.^19^,^21^,^23^

**Fig. 6:**
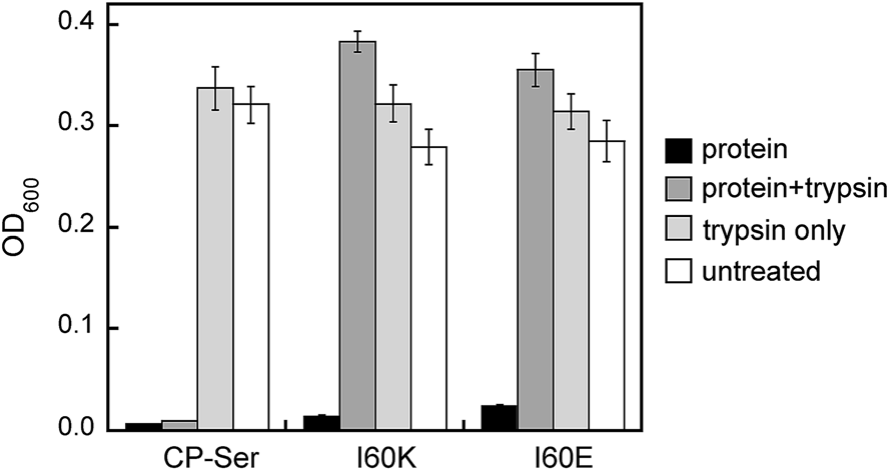
Antimicrobial activity assays performed with untreated and trypsin-treated CP-Ser, I60K, and I60E against *E. coli* ATCC 25922. The OD_600_ values were recorded at *t* = 20 h (mean ± SEM, *n* = 3).

The S100A8 and S100A9 subunits have multiple cleavage sites for the host proteases evaluated in this work (Fig. S1†), and our proteolysis studies revealed that Ca(ii)-bound I60E and I60K were degraded into many proteolytic fragments by these enzymes (Fig. S11–S13†). We therefore ascertained how pre-incubation with trypsin impacts the antibacterial activity of CP-Ser, I60E, and I60K against *E. coli* ATCC 25922. Samples of CP-Ser, I60E, and I60K with and without trypsin were prepared in an antimicrobial activity assay buffer supplemented with Ca(ii), incubated at 37 °C for ≈20 h, and subsequently employed in antimicrobial activity assays. Under standard conditions using the assay medium employed in this work, CP-Ser exhibits full growth inhibition of *E. coli* 25922 at 250 μg mL^–1^.^19^ For the antibacterial activity assays including protease, we employed a final protein concentration of 500 μg mL^–1^. The untreated samples exhibited antimicrobial activity, demonstrating that CP-Ser, I60E, and I60K were stable and active after the overnight incubation (Fig. 6). Trypsin-treated CP-Ser retained the same antimicrobial activity as untreated CP-Ser, whereas trypsin-treated I60E and I60K provided slight growth enhancement relative to the untreated and trypsin-only controls (Fig. 6). HPLC analysis of the protein samples following the overnight incubation with trypsin revealed that ≈50% of CP-Ser remained, whereas I60E and I60K were completely digested (Fig. S15†). The latter result indicates ≈250 μg mL^–1^ of CP-Ser is present when the antimicrobial activity assay was initiated.

### A staphylococcal protease cleaves the S100A8 subunit in the absence of Zn(ii)

**Fig. 7:**
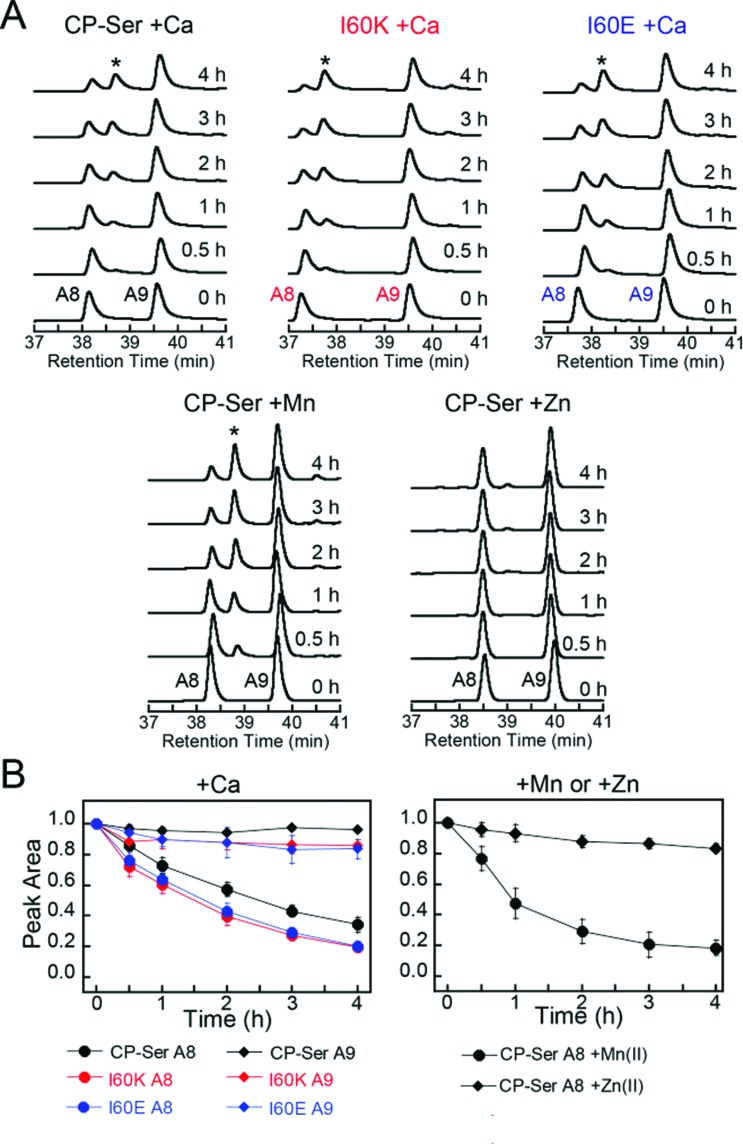
Susceptibility of Ca(ii)-bound CP-Ser, I60E, and I60K (30 μM) to degradation by staphylococcal GluC (0.3 μM). (A) Representative HPLC traces illustrating the full-length S100A8 and S100A9 subunits following incubation with GluC for 0–4 h at 37 °C (75 mM HEPES, 100 mM NaCl, ±1.5 mM CaCl_2_, ±30 μM MnCl_2_, ±60 μM ZnCl_2_, pH 7.5). The asterisk denotes the ΔSHKE truncation product. (B) Reduction in the S100A8 and S100A9 integrated peak areas (normalized to initial peak areas) as a function of time (mean ± SDM, *n* = 3). Full chromatograms are given in Fig. S16.†

Many bacterial pathogens produce extracellular proteases that are capable of degrading proteins central to the immune response.^52^–^54^ To evaluate whether CP is a substrate for an extracellular serine protease produced by a human pathogen, we selected staphylococcal glutamyl endoproteinase (GluC) as an initial case study. This protease is readily available from commercial sources because of its specificity for cleaving after Asp and Glu, which makes it useful for protein mass spectrometry. Moreover, GluC may be a virulence factor^55^–^57^ and CP is abundant at sites of *S. aureus* infection,^16^,^58^ which suggests that it will encounter extracellular proteases released by this pathogen. In contrast to the host proteases that more readily degraded Ca(ii)-bound heterodimers, GluC exhibited no preference for either Ca(ii)-bound CP-Ser or the I60E and I60K variants; each substrate was cleaved in a similar manner (Fig. 7A and S16†). Moreover, the HPLC traces revealed that the presence of GluC results in negligible change to the S100A9 peak (39.8 min) and loss of the S100A8 peak (38.4 min) with concomitant formation of one new peak at 38.7 min (Fig. 7A). LCMS analysis of this new species revealed that GluC cleaves S100A8 between Glu89 and Ser90, resulting in a truncated S100A8 where the last four C-terminal residues (Ser90-His91-Lys92-Glu93) are removed (*m*/*z* calc. 10 336.9 Da, obs. 10 337.3 Da).

The C-terminal region of S100A8 includes His83 and His87, residues that comprise the His_3_Asp metal-binding site, and the GluC cleavage site is in close proximity to His87 (Fig. S1†). Moreover, crystallographic studies of CP-Ser revealed that the S100A8 C-terminus is disordered in the absence of a bound metal at the His_3_Asp site.^22^ The His_3_Asp site coordinates Zn(ii) with high affinity, and we questioned whether Zn(ii) coordination at this site would afford GluC resistance (Fig. 7A). Because each CP heterodimer has two high-affinity Zn(ii) sites,^17^,^19^,^21^ we performed GluC degradation assays with CP-Ser pre-incubated with two equivalents of Zn(ii). Under these conditions, both S100A8 and S100A9 HPLC peaks exhibited negligible change and the S100A8 cleavage product was not observed (Fig. 7A). To confirm that Zn(ii) coordination to the His_3_Asp site provided protection against GluC, we took advantage of the different properties of the two transition metal binding sites and performed an experiment where the protein substrate was pre-incubated with one equivalent of Mn(ii). Because the Mn(ii) affinities of the His_3_Asp and His_6_ vary substantially and Mn(ii) preferentially populates the His_6_ site, the His_3_Asp site remains vacant when CP-Ser (+Ca) is incubated with one equivalent of Mn(ii).^20^,^22^ In these +Mn(ii) experiments, GluC cleaved S100A8 between Glu89 and Ser90 (Fig. 7A). Likewise, the addition of Mn(ii) to the I60E and I60K variants afforded no protection of the GluC cut site (Fig. S16†).

### The GluC degradation product exhibits *in vitro* antibacterial activity

To evaluate the consequences of GluC truncation of the S100A8 subunit, we overexpressed the S100A8 truncation product identified from the GluC digests and the purified S100A8(C42S) (S90Stop)/S100A9 heterodimer, abbreviated ΔSHKE herein. This CP-Ser variant exhibits the expected α-helical fold and forms heterotetramers in the presence of Ca(ii) (Fig. S3 and S17†). Antibacterial activity assays employing *E. coli* ATCC 25922, *S. aureus* ATCC 25923, and *Lactobacillus plantarum* WCFS1 revealed that ΔSHKE exhibits the same *in vitro* growth inhibitory activity as CP-Ser against each strain (Fig. S18†). Thus, the last four residues of the S100A8 C-terminus are not essential for *in vitro* antibacterial activity of human CP against these three strains. We selected these strains on the basis of metabolic metal requirements and how the metal-binding sites of CP-Ser contribute to *in vitro* growth inhibition.^19^,^21^,^24^ Prior studies showed that the His_6_ site, which sequesters Mn(ii), Fe(ii), and Zn(ii), contributes more to growth inhibition of *E. coli* and *S. aureus* than the His_3_Asp site under these assay conditions.^19^,^21^,^23^,^24^ The His_3_Asp site sequesters Zn(ii) but not Fe(ii) or Mn(ii), and this site is essential for antibacterial activity against *L. plantarum*.^24^ This microbe has no metabolic iron requirement and is sensitive to Zn(ii) restriction under the assay conditions; thus, growth studies with *L. plantarum* allow for interrogation of the His_3_Asp site. The fact that the ΔSHKE variant showed full growth inhibitory activity against *L. plantarum* indicated that loss of the four C-terminal residues of S100A8 does not impair the ability of the His_3_Asp site to sequester Zn(ii) from this organism. Moreover, these *in vitro* data suggest that GluC cleavage of S100A8 is not an effective staphylococcal resistance mechanism against human CP, although it is possible that synergism with other factors occurs in the complex milieu of an infection site. In light of these observations, we note that the C-terminus of human S100A8 differs from the C-termini of other mammalian orthologues. Human S100A8 is four residues longer than other sequenced mammalian S100A8 polypeptides, and these additional four residues occur in the C-terminal region. The C-terminal residues 87–93 of human S100A8 are HEESHKE, and the C-terminal residues 87–89 of other mammalian S100A8 are HKE. Whether this difference has functional significance for human CP is unclear.

### Biological implications and outlook

This work provides the first comprehensive evaluation of how metal binding and changes in quaternary structure influence the proteolytic stability of human CP. Our results indicate that Ca(ii)-induced heterotetramerization and Zn(ii) chelation enable CP to resist degradation by select host and bacterial serine proteases. We reason that the metal-induced structural changes enable CP to persist in the protease-rich extracellular space and thereby function in metal withholding and the inflammatory response. It will be important to determine whether these observations pertain to the fate of CP in the presence of other proteases, particularly those employed by bacterial pathogens to thwart the innate immune response. Although the current data indicate that CP exhibits remarkable metal-dependent resistance to the proteases evaluated in this work, pathogens overcome the host response to infection. We therefore expect that certain pathogenic microbes deploy degradation machinery that can disarm CP. Another important avenue for future inquiry is to consider the current observations in the context of biological concentrations, regulation, and other factors that come into play in the variable and dynamic extracellular environments of infection and inflammatory sites. In broad terms, this work provides an additional and previously unappreciated facet to how Ca(ii) ions influence CP function, and further exemplifies the fact that CP is a complex and multifaceted protein and that its functional properties will be governed by its speciation.

## Experimental section

### General materials and methods

All solvents, reagents, and chemicals were obtained from commercial suppliers and used as received. All buffers and metal solutions were prepared using Milli-Q water (18.2 MΩ cm, 0.22 μm filter, Millipore). For metal-binding experiments, HEPES buffer was prepared with Ultrol grade HEPES (free acid, Calbiochem) and TraceSELECT NaCl (Fluka), and aqueous TraceSELECT NaOH (Sigma) was used for pH adjustments. Disposable polypropylene spatulas were used to transfer buffer components. Buffers were treated with Chelex 100 resin (BioRad, 10 g L^–1^) by stirring each buffer/Chelex mixture in a polypropylene beaker for at least 1 h, and the Chelex was removed by filtration through a 0.22 μm filter. All buffers were stored in polypropylene bottles. A Tris buffer (1 mM Tris, 0.5 mM EDTA, pH 8.5) was prepared from Tris base (J. T. Baker) and EDTA disodium dihydrate (Mallinckrodt) and used for circular dichroism spectroscopy. Calcium chloride (99.99%) and manganese chloride (99.99%) were purchased from Alfa Aesar. Anhydrous zinc chloride (99.999%) and ferrous ammonium sulfate (99.997%, Fe(NH_4_)_2_(SO_4_)_2_·6H_2_O or Mohr's salt) were purchased from Sigma. Stock solutions of CaCl_2_ (370 mM), MnCl_2_ (1 M), and ZnCl_2_ (100 mM) were prepared by using Milli-Q water and acid-washed volumetric glassware, and were stored in polypropylene tubes. For preparation of the Fe(ii) stock solution (100 mM), the Fe(ii) salt was weighed on the bench top, transferred to a polypropylene tube, and then brought into a glove box (Vacuum Atmospheres Company, nitrogen atmosphere). The iron salt was dissolved in Milli-Q water that was degassed with Ar and stored in the glove box. All subsequent iron-containing samples were prepared in the glove box using buffers that were degassed with Ar and stored in the glove box. Working solutions of metals were prepared fresh for each experiment by diluting the stock solution into buffer (75 mM HEPES, 100 mM NaCl, pH 7.5) or Milli-Q water. Protein concentration was determined by optical absorbance at 280 nm using a BioTek Synergy HT plate reader outfitted with a calibrated Take3 Micro-Volume plate, and the appropriate extinction coefficient (Table S2†).

### Instrumentation

An Agilent 1200 series instrument equipped with a thermostatted column compartment set to 20 °C, and a multi-wavelength detector set at 220 and 280 nm (500 nm reference wavelength with 100 nm bandwidth), was used to perform analytical high-performance liquid chromatography (HPLC). A Proto C4 column (5 μm pore, 4.6 × 250 mm, Higgins Analytical Inc.) set at a flow rate of 1 mL min^–1^ was employed for all analytical HPLC experiments. HPLC-grade acetonitrile (MeCN) and trifluoroacetic acid (TFA) were routinely purchased from EMD and Alfa Aesar, respectively. For all HPLC runs, solvent A was 0.1% TFA/H_2_O and solvent B was 0.1% TFA/MeCN.

An Agilent 1260 series LC system equipped with an Agilent 6230 TOF system housing Agilent Jetstream ESI source was employed to perform high-resolution mass spectrometry. An Agilent Poroshell 300SB-C18 (5 μm pore) and denaturing protocol were utilized for all LC-MS analyses. Solvent A was 0.1% formic acid/H_2_O. Solvent B was 0.1% formic acid/MeCN. Protein samples (5 μM) were prepared in water and 1 μL was injected for each analysis. The S100A8 and S100A9 subunits were eluted by using a gradient 0–65% over 30 min. The resulting mass spectra were deconvoluted using the maximum entropy algorithm in MassHunter BioConfirm (Agilent).

The optical density (OD_600_) of bacterial cultures and optical absorption spectroscopy were carried out with a Beckman Coulter DU 800 spectrophotometer thermostatted at 25 °C with a Peltier temperature controller. Fluorescence spectra were collected on a Photon Technologies International QuantaMaster 40 fluorimeter outfitted with a continuous xenon source for excitation, autocalibrated QuadraScopic monochromators, a multimode PMT detector, and a circulating water bath maintained at 25 °C. This instrument was controlled by the FelixGX software package. FelixGX was used to integrate the emission spectra.

### Protein purification

Variants of human calprotectin were overexpressed and purified as described previously.^19^ All variants are based on CP-Ser, which is comprised of S100A8(C42S) and S100A9(C3S), and the protocol affords the S100A8/S100A9 heterodimer form of each variant. Protein yields for the variants I60E, I60K, and ΔSHKE ranged from 12 to 35 mg L^–1^ of culture. The purified proteins were stored at –80 °C, and only thawed once immediately before use.

### Site-directed mutagenesis

A modified Quick-Change site-directed mutagenesis protocol was employed to generate plasmids encoding S100A8(C42S)(I60K), S100A8(C42S)(I60E), and S100A8(C42S)(ΔSHKE). The template plasmids and primers are listed in Table S1.† PCR amplification was carried out using PfuTurbo DNA polymerase. For the I60K and I60E mutants, the PCR protocol was: 95 °C for 30 s, 95 °C for 30 s, 55 °C for 1 min, 68 °C for 15 min, (25 cycles), and 4 °C hold. For the ΔSHKE mutant, the protocol was the same, but the annealing temperature was 51 °C. After PCR amplification, the template DNA was digested by DpnI (New England Biolabs) by adding 1 μL of the restriction enzyme to a 25 μL PCR reaction at *t* = 0 and 1.5 h with incubation at 37 °C. The digestion products were transformed into chemically competent *E. coli* TOP10 cells. Overnight cultures (5 mL, 50 μg mL^–1^ kanamycin) were grown from single colonies. The plasmids were isolated using a miniprep kit (Qiagen). The presence of the mutations and fidelity of the protein coding sequences were verified by DNA sequencing (Quintara Biosciences).

### Analytical size exclusion chromatography

An ÄKTA purifier (GE Lifesciences) housed in a 4 °C cold room and outfitted with a 100 μL sample loop was used to perform all analytical size exclusion chromatography (SEC) experiments. A Superdex 75 10/300 GL column (GE Lifesciences) equilibrated in running buffer was calibrated with a low-molecular-weight calibration mixture (GE Lifesciences) as described previously.^19^ The protein of interest was thawed at room temperature and buffer exchanged from the storage buffer into the running buffer using a filter (0.5 mL, 10 kDa MWCO, Amicon), and the protein concentration was adjusted to 30 μM by diluting with the running buffer. For the experiments with Ca(ii), 600 μM CaCl_2_ was included in the running buffer and protein sample. For experiments with Mn(ii) or Fe(ii) only, 300 μM MnCl_2_ or Fe(NH_4_)_2_(SO_4_)_2_·6H_2_O was added to the sample only. For experiments with both Ca(ii) and Mn(ii) or both Ca(ii) and Fe(ii), the running buffer and sample contained 600 μM CaCl_2_ and 33 μM MnCl_2_ or 33 μM Fe(NH_4_)_2_(SO_4_)_2_·6H_2_O was included in the sample only. Samples were incubated for 15 min at 4 °C after adding metals and then centrifuged at 13 000 rpm for 10 min. The entire volume of each sample (30 μM protein, 300 μL) was loaded onto the 100 μL sample loop. The loop was emptied with 0.5 mL of running buffer, and the protein was eluted over one column volume at a flow rate of 0.5 mL min^–1^ at 4 °C. Fe(ii)-containing samples were allowed to incubate for 50 min in the glove box before being centrifuged at 13 000 rpm for 10 min.

### Sedimentation velocity experiments

A Beckman XL-I Analytical Ultracentrifuge outfitted with an An-60 Ti rotor was employed for all sedimentation velocity (SV) experiments. The rotor housed conventional double-sector charcoal filled Epon centerpieces within the sample cells and contained quartz (absorption optics) or sapphire (interference optics) windows. The absorption wavelength for optical detection was 280 nm. The samples were centrifuged at 42 000 rpm and 20 °C until sedimentation was complete. SEDNTERP^59^ was employed to calculate the buffer viscosity (*η*), buffer density (*ρ*), and protein partial specific volume (*v*) at 20 °C. The sedimentation velocity data was analyzed using previously described methods,^60^ and full details are provided as ESI.† Hydrodynamic modeling computations were performed with HYDROPRO^44^ using the crystal structures of Ca(ii)-bound CP-Ser (PDB ; 1XK4 36) and Mn(ii)-, Ca(ii)-, and Na(i)-bound CP-Ser (PDB ; 4XJK 22) to obtain theoretical sedimentation coefficients for the CP-Ser heterodimer and heterotetramer. All modeling was carried out with buffer viscosity (*η*) and buffer density (*ρ*) of water at 20 °C and a protein partial specific volume (*v*) of 0.7388 mL g^–1^.

One day prior to an experiment, each protein sample was thawed and diluted to 27 μM in 75 mM HEPES, 100 mM NaCl at pH 7.5. The resulting samples were dialyzed against 1 L of the same buffer containing 10 g of Chelex resin at 4 °C overnight. The dialyzed samples were transferred to 1.7 mL polypropylene tubes and centrifuged (13 000 rpm, 5 min, 4 °C) to sediment any Chelex resin. Aliquots of the dialysis buffer were centrifuged (3000 rpm, 5 min, 4 °C) in 50 mL polypropylene tubes and the supernatant was used for the reference samples in the SV experiment. In select experiments, EDTA (30 μM), CaCl_2_ (540 μM), and/or MnCl_2_ (30 μM) were added to the reference and protein samples. The references and protein samples were allowed to incubate while the SV window assemblies were constructed (≈1.5 h). The SV window assemblies were loaded with 410 μL of reference buffer and 400 μL of protein containing sample.

### Protease digestion assays

Trypsin (Affymetrix) and chymotrypsin (Amresco) were obtained as lyophilized powders, stored at 4 °C, and dissolved in water to afford solutions of ≈50–100 μM immediately before use. Reported extinction coefficients for trypsin and chymotrypsin were used to determine the concentration (Table S2†).^61^,^62^ Frozen stock solutions of glutamyl endopeptidase (GluC) (New England Biolabs), and human neutrophil elastase (HNE) (Enzo Life Sciences) were obtained as lyophilized powders. The entire portion of each protease was dissolved in assay buffer to afford solutions with concentrations of ≈0.1 mg mL^–1^ and ≈1 mg mL^–1^ for GluC and HNE, respectively. The solutions were stored at –20 °C (GluC) or –80 °C (HNE). Protease digestion assays were performed on a 350 μL scale at pH 7.5 (75 mM HEPES, 100 mM NaCl). Aliquots of CP-Ser, I60E, and I60K were thawed at room temperature and diluted to 30 μM using the assay buffer in 1.7 mL microcentrifuge tubes. To select samples, calcium chloride (1.5 mM), manganese chloride (30 μM), and zinc chloride (60 μM) were added, and the resulting solutions were incubated at room temperature for at least 15 min prior to the assay. To initiate each digestion assay, an aliquot (between 5 and 10 μL as appropriate) of protease was added to the 350 μL the protein solution to bring the protease concentration to 0.45 μM (trypsin) or 0.3 μM (others). The resulting solution was immediately mixed with a pipet and incubated at 37 °C. Aliquots (45 μL) of the reaction were quenched with 155 μL of aqueous 0.77% (v/v) TFA at *t* = 0, 30 min, 1, 2, 3, and 4 h. The quenched solutions were centrifuged (13 000 rpm, 10 min, 4 °C), and the resulting samples were analyzed by analytical HPLC using a solvent gradient of 10–60% B over 50 min. S100A8 eluted at ≈38 min (depending on the mutant) and S100A9 eluted at 39.8 min. Select peaks were collected manually and further analyzed by LC-MS. Controls without protease were prepared, quenched, and analyzed in an identical manner except that ubiquitin (Sigma) was added to 0.3 μM. Frozen stocks of ubiquitin (117 μM, 1 mg mL^–1^) were prepared in water, stored at –20 °C, and thawed before use. Experimental details for control studies to confirm that the divalent cations do not affect protease activity are provided in Fig. S19–S22.†

### Antimicrobial activity assays

The growth inhibitory activities of CP-Ser, I60K, I60E, and ΔSHKE against *Escherichia coli* ATCC 25922, *Staphylococcus aureus* ATCC 25923 and *Lactobacillus plantarum* WCFS1 (CP-Ser and ΔSHKE only) were assayed at 30 °C as described previously.^19^,^24^ The antimicrobial activity assay medium, hereafter AMA medium, was a 62 : 38 ratio of 20 mM Tris–HCl, pH 7.5, 100 mM NaCl, 5 mM BME, 3 mM CaCl_2_ and tryptic soy broth (TSB) with 0.25% (w/v) dextrose. For *L. plantarum*, de Man, Rogosa and Sharpe (MRS) broth (CRITERION) without additional dextrose was used in place of TSB. To prevent evaporation of the medium, the plates were sealed with parafilm and a beaker of water was housed in the incubator shaker.

### Antimicrobial activity assay with trypsin

The growth inhibitory activities of CP-Ser, I60E, and I60K pre-incubated with trypsin (Affymetrix) were assayed by modifying a literature protocol for standard antimicrobial activity assays.^19^ Protein aliquots were thawed at room temperature and buffer exchanged into AMA buffer (20 mM Tris–HCl, 100 mM NaCl, 3 mM CaCl_2_, pH 7.5) three times using spin filter (0.5 mL, 10 kDa MWCO, Amicon) that were sterilized by exposure to UV light for 15 min. For each protein, two aliquots (233 μM, 45 μL) were prepared. Trypsin (≈2 mg) was dissolved in AMA buffer, the trypsin concentration was determined using the reported extinction coefficient (*ε*_280_ = 30 000 M^–1^ cm^–1^, Table S2†)^61^ and the sample was diluted to 4.5 μM using AMA buffer. For each set of 45 μL protein aliquots, a 5 μL aliquot of trypsin was added to one and a 5 μL aliquot of AMA buffer to the other. A trypsin-only solution was prepared that contained 0.45 μM trypsin and no protein substrate. The solutions were incubated at 37 °C for ≈20 h and subsequently used in the antibacterial activity assay.

*E. coli* ATCC 25922 was inoculated into 5 mL of TSB containing 0.25% (w/v) dextrose and grown overnight at 37 °C in a rotating wheel (≈16 h). The culture was diluted 1 : 100 into 5 mL of fresh TSB with (0.25% w/v) dextrose and grown with shaking (37 °C, ≈2 h) until the OD_600_ reached 0.6. The culture was diluted 1 : 500 into fresh AMA medium. The antibacterial activity assay was carried out in sterile polystyrene 96-well plates (Corning). Each well contained 10 μL of the digested protein, undigested protein (no protease control), trypsin only, or buffer only and 90 μL of the diluted bacterial culture. Control wells containing 10 μL of trypsin and 90 μL of sterile AMA medium was also included, and no bacterial growth was observed in these wells. The final concentration of each CP-Ser sample was 500 μg mL^–1^ (21 μM), and previous work has shown that CP-Ser inhibits growth of *E. coli* and *S. aureus* at this concentration.^19^,^21^ Each condition was set up in triplicate. Each plate was sealed with parafilm and incubated at 30 °C with shaking at 150 rpm in a tabletop incubator-shaker containing a beaker filled with water. Bacterial growth was monitored by OD_600_ values, which were measured at several time points (0 to 20 h) by using a plate reader (BioTek). Three independent replicates were conducted on different days. Different *E. coli* freezer stocks were used for each replicate, and at least two independent medium preparations were employed, and two different preparations of each protein were used over the triplicate. The resulting averages and standard errors of the mean are reported (*n* = 9).

### Protease activity assays

To determine if the metals used in the digestion assays altered protease activity, we carried out activity assays with small molecule substrates. Protease stock solutions were prepared as described above. All experiments were carried out in triplicate and averaged. For trypsin, *N*_α_-benzoyl-l-arginine ethyl ester HCl (BAEE, Santa Cruz Biotech) was dissolved to 10 mM in 75 mM HEPES, 100 mM NaCl, pH 7.5, and then diluted to 80 μM in the same buffer with 1.5 mM CaCl_2_ with or without 30 μM MnCl_2_. These solutions were dispensed into quartz cuvettes (Starna), 1 mL of solution per cuvette. The reaction was initiated by adding trypsin to 8.4 nM in a cuvette and briefly shaking. The reaction was monitored continuously at 253 nm. An analogous experiment was carried out to assay the activities of chymotrypsin and human neutrophil elastase (HNE). For chymotrypsin, the substrate *N*-succinyl-Ala-Ala-Pro-Phe-*p*-nitroanilide (Enzo Life Sciences) was dissolved to 40 mM in dimethylformamide. In the experiment, the chymotrypsin was diluted to 2 nM and the substrate was at 0.4 mM. The chymotrypsin reactions were continuously monitored at 410 nm in quartz cuvettes. For HNE, the substrate *N*-succinyl-Ala-Ala-Val-Ala-*p*-nitroanilide (Santa Cruz Biotech) was dissolved to 100 mM dimethylsulfoxide. In the assay, HNE was diluted to 10 nM, and the substrate was at 0.5 mM. The HNE reactions were continuously monitored at 410 nm in polystyrene cuvettes.

The activity of glutamyl endopeptidase (GluC) was assayed with carboxybenzyl-Leu-Leu-Glu-β-naphthylamide. A 1 mM stock solution of the substrate was made in dimethylsulfoxide. The reactions were carried out on a 1 mL scale in quartz cuvettes (Starna), and monitored by fluorescence emission. In the assays with Ca(ii) or Mn(ii), the substrate was diluted to 100 μM. For the assays with Zn(ii), CP-Ser (30 μM) and Zn(ii) (60 μM) were combined in the assay buffer and the GluC substrate was added last. This modification was made because precipitation occurred when Zn(ii) was introduced into solutions containing the substrate. The reactions were initiated by adding GluC to 67 nM. The reaction was monitored with all slits set to 0.8 mm, excitation at 340 nm, and scanning from 400–430 nm at a rate of 0.1 nm s^–1^. The progress of the reaction was determined by monitoring the fluorescence emission at 410 nm.

### Mn(ii) competition experiments with ZP1

Competition between CP variants and ZP1 for Mn(ii) was assayed as previously described except that 1 μM ZP1 and 4 μM protein were employed.^20^ Experiments to measure the number of equivalents of Ca(ii) required for CP variants to outcompete ZP1 for Mn(ii) were performed as previously described, but the ZP1 and MnCl_2_ concentrations were 1 μM and 3.5 μM, respectively.^20^ Averages and the standard deviations are reported for both experiments (*n* = 3).

### Circular dichroism spectroscopy

An Aviv Model 202 circular dichroism (CD) spectrometer thermostatted at 25 °C was employed for CD spectroscopy. A 1 mm path-length CD cell (Hellma) was employed for all CD measurements. All protein samples (10 μM protein, 300 μL, 1 mM Tris, 0.5 mM EDTA, pH 8.5, ±2 mM CaCl_2_) were made at the same time before beginning data acquisition. Wavelength scans were carried out from 190–260 nm with 1 nm steps (5 s averaging time, three averaged scans).

## Supplementary Material

Supplementary information
