# Supplementary material for: Calcium-induced tetramerization and zinc chelation shield human calprotectin from degradation by host and bacterial extracellular proteases
Source: Chem Sci. 2015 Nov 23;7(3):1962–75. doi: 10.1039/c5sc03287c (PMC4763987; doi:10.1039/c5sc03287c)
Supplement: Supplementary file 1 [file SC-007-C5SC03287C-s001.pdf]

Electronic Supplementary Material (ESI) for Chemical Science.
This journal is © The Royal Society of Chemistry 2015

Supplementary Information for

Calcium-induced Tetramerization and Zinc Chelation Shield Human Calprotectin from Degradation by Host and Bacterial Extracellular Proteases

Jules R. Stephan and Elizabeth M. Nolan*

Department of Chemistry, Massachusetts Institute of Technology, Cambridge, MA 02139, USA

*Corresponding author: lnolan@mit.edu

Phone: 617-452-2495

Fax: 617-324-0505

This Supplementary Information section includes:

Supplementary Table and Figures………………………………………………………… S3
Table S1. Primers for tetramer-deficient mutants and GluC cleavage product.................. S3
Table S2. Extinction coefficients and molecular weights of proteins.................................. S4
Table S3. Mass spectrometric analysis of CP-Ser and variants.. S4
Table S4. SEC elution volumes and calculated molecular weights.................................... S5
Table S5. SEDFIT sedimentation coefficients for CP-Ser and tetramer-deficient variants.. S6
Table S6. DCDT+ sedimentation coefficients for CP-Ser and tetramer-deficient variants... S7
Fig. S1. Amino acid sequences and potential protease cleavage sites in CP-Ser.............. S8
Fig. S2. SDS-PAGE of CP-Ser and varaints.. S9
Fig. S3. CD spectra of CP-Ser and variants.. S10
Fig. S4. Mn(II) competition titrations of CP-Ser and variants………................................ S11
Fig. S5. Mn(II) competition titrations of CP-Ser and variants with addition of Ca(II)……..... S12
Fig. S6. SEC of CP-Ser and tetramer-deficient variants with Mn(II).................................. S13
Fig. S7. SEC of CP-Ser and tetramer deficient variants with Fe(II).................................... S14
Fig. S8. SEDFIT sedimentation distributions and residuals bitmaps................................. S15
Fig. S9. DCDT+ sedimentation distributions and residuals plots…………………………….. S16
Fig. S10. HPLC traces and LCMS of undigested CP-Ser and tetramer-deficient mutants... S17
Fig. S11. Full HPLC chromatograms of CP-Ser, I60K, and I60E trypsin digestions........... S18
Fig. S12. Full HPLC chromatograms of CP-Ser, I60K, and I60E chymotrypsin digestions.. S19
Fig. S13. Full HPLC chromatograms of CP-Ser, I60K, and I60E HNE digestions…………. S20
Fig. S14. Antibacterial activity of CP-Ser, I60K, and I60E………………......................... S21
Fig. S15. HPLC chromatograms of trypsin-treated and untreated proteins for AMAs.......... S22
Fig. S16. Full HPLC chromatograms of CP-Ser, I60K, and I60E GluC digestions.............. S23
Fig. S17. SEC of CP-Ser and ∆SHKE with and without Ca(II).. S24
Fig. S18. Antibacterial activity of CP-Ser and ∆SHKE... S25
Fig. S19. Trypsin activity assay... S26
Fig. S20. Chymotrypsin activity assay.. S27
Fig. S21. Human neutrophil elastase activity assay.. S28
Fig. S22. Glutamyl endopeptidase activity assay.. S29
Supplementary References………………………………………………………………….. S30

Table S1. Primers employed for site-directed mutagenesis.[a]

Primer	Sequence[b]
I60K-1	5'-GTTTAAGGAGTTGGAC**AAG**AACACGGATGGCGCTG-3'
I60K-2	5'-CAGCGCCATCCGTGTT**CTT**GTCCAACTCCTTAAAC-3'
I60E-1	5'-GTTTAAGGAGTTGGAC**GAA**AACACGGATGGCGCTG-3'
I60E-2	5'-CAGCGCCATCCGTGTT**TTC**GTCCAACTCCTTAAAC-3'
ΔSHKE-1[c]	5'-GAAGAGCCACGAAGAG**TAA**CATAAAGAGTAACTC-3'
ΔSHKE-2[c]	5'-GAGTTACTCTTTATG**TTA**CTCTTCGTGGCTCTTC-3'

[a] pET41a-*S100A8(C42S)* was employed as the template plasmid. This plasmid has the S100A8(C42S) gene inserted between the *Nde*I and *Xho*I restriction sites.[S1] [b] The codons containing mutations are underlined and colored red. [c] These primers provide a stop codon at position 90 (S90Stop).

Table S2. Molecular weights and extinction coefficients for proteins used in this study.

Protein	Molecular Weight (Da)[a]	ε_{280} (M^{-1} cm^{-1})[b]
A8(C42S)	10 818.5	11 460
A8(C42S)(I60K)	10 833.5	11 460
A8(C42S)(I60E)	10 834.5	11 460
A8(C42S)(ΔSHKE)	10 336.9	11 460
A9(C3S)	13 094.7[c]	6 990
Trypsin	23 300	30 000[S2]
Chymotrypsin	25 000	50 000[S3]
Glutamyl endopepidase (GluC)	30 000	-
Human neutrophil elastase (HNE)	28 500	-

[a] Molecular weights were calculated by using the ProtParam tool available on the ExPASy server (http://web.expasy.org/protparam). [b] Extinction coefficients (280 nm) were calculated by using the ProtParam tool. [c] In all preparations, LCMS revealed that the dominant purified species lacked the N-terminal methionine. The peptide molecular weight is the theoretical value for S100A9(C3S) lacking the N-terminal Met residue.

Table S3. Mass spectrometric analysis of CP-Ser and variants.

CP-Ser Variant	S100A8 Observed Mass (g/mol)	S100A9 Observed Mass[a] (g/mol)
CP-Ser	10 818.6	13 094.7
I60K	10 833.7	13 095.0
I60E	10 834.7	13 094.9
ΔSHKE	10 337.1	13 095.0

[a] In all preparations, the dominant purified species of the S100A9 subunit lacked the N-terminal methionine. The masses reported here are the observed values for S100A9(C3S) lacking the N-terminal Met residue.

Table S4. Analytical SEC elution volumes and calculated molecular weights.[a]

Ca(II)	Mn(II)	Fe(II)	Protein	Elution Volume (mL)	Calculated Molecular Weight (kDa)
−	−	−	CP-Ser	11.5	34.9
−	−	−	I60K	11.5	34.9
−	−	−	I60E	11.5	34.9
−	−	−	ΔSHKE	11.7	32.2
+	−	−	CP-Ser	10.7	48.2
+	−	−	I60K	11.8	31.0
+	−	−	I60E	11.8	31.0
+	−	−	ΔSHKE	10.9	44.5
−	−	+	CP-Ser	11.0	42.7
−	−	+	I60K	11.0	42.7
−	−	+	I60E	11.1	41.0
+	−	+	CP-Ser	11.0	42.7
+	−	+	I60K	11.1	41.0
+	−	+	I60E	11.1	41.0
−	+	−	CP-Ser	11.0	42.7
−	+	−	I60K	11.5	34.9
−	+	−	I60E	11.5	34.9
+	+	−	CP-Ser	11.1	41.0
+	+	−	I60K	10.9	44.5
+	+	−	I60E	11.1	41.0

[a] Each sample contained 30 μM protein (75 mM HEPES, 100 mM NaCl, pH 7.5). The +Ca(II) samples contained 600 μM Ca(II) in the sample and running buffer. The +Mn(II) samples contained 300 μM Mn(II) in the sample only. In the +Ca(II) +Mn(II) experiments, the running buffer and sample contained 600 μM Ca(II) and only the sample contain 33 μM Mn(II). The experiments were performed at 4 °C.

Table S5. Calculated sedimentation coefficients and molecular weights using Sedfit.[a]

Protein	Concentration (μM)	$s_{20,w}$ (S)	MW (kDa)	Partial Specific Volume (mL/g)
CP-Ser[b]	27.5	2.4	22.7	0.7388
CP-Ser[d]	27.5	3.9	43.5	0.7388
CP-Ser[e]	27.5	2.4 (35%), 4.1 (65%)	20.6, 46.1	0.7388
CP-Ser[f]	27.5	4.5 (85%), 6.5 (15%)	40.8, 70.8	0.7388
I60K[c]	27.5	2.4	23.0	0.7388
I60K[d]	27.5	2.4	24.1	0.7388
I60K[e]	27.5	2.4 (73%), 4.1 (27%)	22.9, 51.3	0.7388
I60K[f]	27.5	4.1 S	43.7	0.7388
I60E[c]	27.5	2.3	22.8	0.7388
I60E[d]	27.5	2.3	24.9	0.7388
I60E[e]	27.5	2.2 (84%), 3.9 (16%)	23.8, 56.0	0.7388
I60E[f]	27.5	4.0	44.9	0.7388

[a] All experiments were conducted at 20 °C. The units of viscosity are in centipoise (cP) (1 Poise g cm^{-1} s^{-1}). Sedimentation coefficients are in Svedbergs (1 Svedberg = 100 fs = 1 x 10^{-13} s). The *c(s)* method was used for fitting the data. All scans that began at the baseline were used in fitting. [b] The sample buffer was 75 mM HEPES, 100 mM NaCl, 1.35 mM EDTA, pH 7.5. $s_{20,w}$ values were adjusted with solvent density (ρ) of 1.00825 g/mL, solvent viscosity (η) of 1.0563 cP, and pH 7.5 at 20 °C. [c] The sample buffer was 75 mM HEPES, 100 mM NaCl, pH 7.5. $s_{20,w}$ values were adjusted with solvent density (ρ) of 1.0082 g/mL, solvent viscosity (η) of 1.0565 cP, and pH 7.5 at 20 °C. [d] The sample buffer was 75 mM HEPES, 100 mM NaCl, 540 μM $CaCl_2$, and pH 7.5. $s_{20,w}$ values were adjusted with solvent density (ρ) of 1.0082 g/mL, solvent viscosity (η) of 1.0565 cP, and pH 7.5 at 20 °C. [e] The sample buffer was 75 mM HEPES, 100 mM NaCl, 27.5 μM $MnCl_2$, pH 7.5. $s_{20,w}$ values were adjusted with solvent density (ρ) of 1.0082 g/mL, solvent viscosity (η) of 1.0565 cP, and pH 7.5 at 20 °C. [f] The sample buffer was 75 mM HEPES, 100 mM NaCl, 540 μM $CaCl_2$, 27.5 μM $MnCl_2$, pH 7.5. $s_{20,w}$ values were adjusted with solvent density (ρ) of 1.0082 g/mL, solvent viscosity (η) of 1.0565 cP, and pH 7.5 at 20 °C.

Table S6. Calculated sedimentation coefficients and molecular weights using DCDT+.[a]

Protein	Concentration (μM)	$s_{20,w}$ (S)	D (F)	MW (kDa)	Partial Specific Volume (mL/g)
CP-Ser[b]	27.5	2.4	12.0	18.8	0.7388
CP-Ser[d]	27.5	3.8	7.80	45.3	0.7388
CP-Ser[e]	27.5	3.6	10.7	31.2	0.7388
CP-Ser[f]	27.5	4.8	12.5	35.6	0.7388
I60K[c]	27.5	2.3	11.8	18.7	0.7388
I60K[d]	27.5	2.6	13.3	17.9	0.7388
I60K[e]	27.5	2.8	12.3	13.5	0.7388
I60K[f]	27.5	4.1	8.9	42.8	0.7388
I60E[c]	27.5	2.4	10.8	20.9	0.7388
I60E[d]	27.5	2.5	10.8	22.3	0.7388
I60E[e]	27.5	2.7	13.4	18.8	0.7388
I60E[f]	27.5	4.0	8.4	44.4	0.7388

[a] All experiments were conducted at 20 °C. The units of viscosity are in centipoise (cP) (1 Poise g cm^{-1} s^{-1}). Sedimentation coefficients are in Svedbergs (1 Svedberg = 100 fs = 1 x 10^{-13} s). Diffusion coefficients correspond to the best-fit molecular mass in Fick units (1 Fick = 1 x 10^{-7} cm^2/s. The dc/dt method was used for all data except the following: CPSer+Ca(II) where *g(s*)* was used. The typical scan range was 14-21 and the peak broadening limit was always greater than 60 kDa. For the manganese samples, only six scans were used. [b] The sample buffer was 75 mM HEPES, 100 mM NaCl, 1.35 mM EDTA, pH 7.5. $s_{20,w}$ values were adjusted with solvent density (ρ) of 1.00825 g/mL, solvent viscosity (η) of 1.0563 cP, and pH 7.5 at 20 °C. [c] The sample buffer was 75 mM HEPES, 100 mM NaCl, pH 7.5. $s_{20,w}$ values were adjusted with solvent density (ρ) of 1.0082 g/mL, solvent viscosity (η) of 1.0565 cP, and pH 7.5 at 20 °C. [d] The sample buffer was 75 mM HEPES, 100 mM NaCl, 540 μM $CaCl_2$, and pH 7.5. $s_{20,w}$ values were adjusted with solvent density (ρ) of 1.0082 g/mL, solvent viscosity (η) of 1.0565 cP, and pH 7.5 at 20 °C. [e] The sample buffer was 75 mM HEPES, 100 mM NaCl, 27.5 μM $MnCl_2$, pH 7.5. $s_{20,w}$ values were adjusted with solvent density (ρ) of 1.0082 g/mL, solvent viscosity (η) of 1.0565 cP, and pH 7.5 at 20 °C. [f] The sample buffer was 75 mM HEPES, 100 mM NaCl, 540 μM $CaCl_2$, 27.5 μM $MnCl_2$, pH 7.5. $s_{20,w}$ values were adjusted with solvent density (ρ) of 1.0082 g/mL, solvent viscosity (η) of 1.0565 cP, and pH 7.5 at 20 °C.

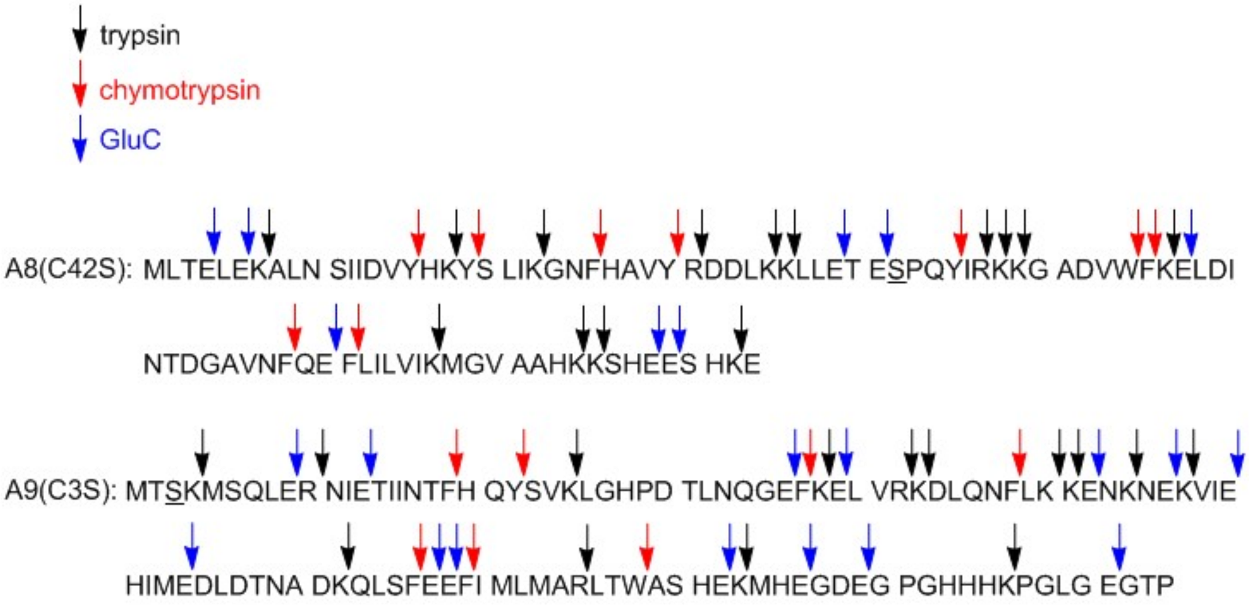

Fig. S1 Amino acid sequences and potential cleavage sites in human S100A8(C42S) and S100A9(C3S) for trypsin, chymotrypsin (restricted to aromatic amino residues), and GluC (restricted to glutamate residues). Residues mutated from Cys to Ser are underlined. Both subunits have at least seven potential cleavage sites for each protease.

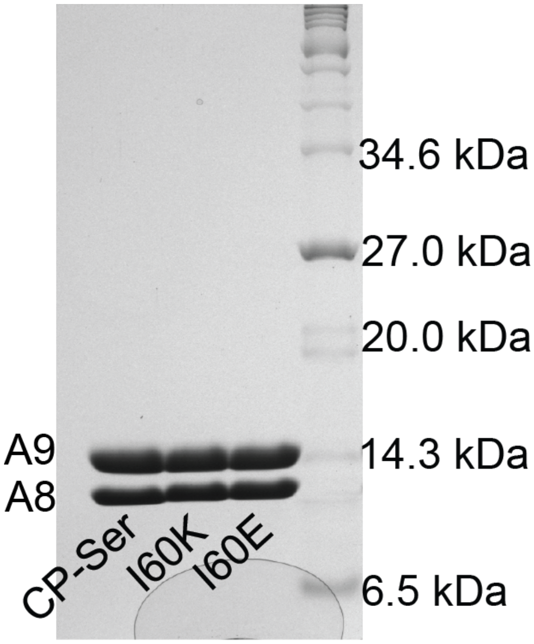

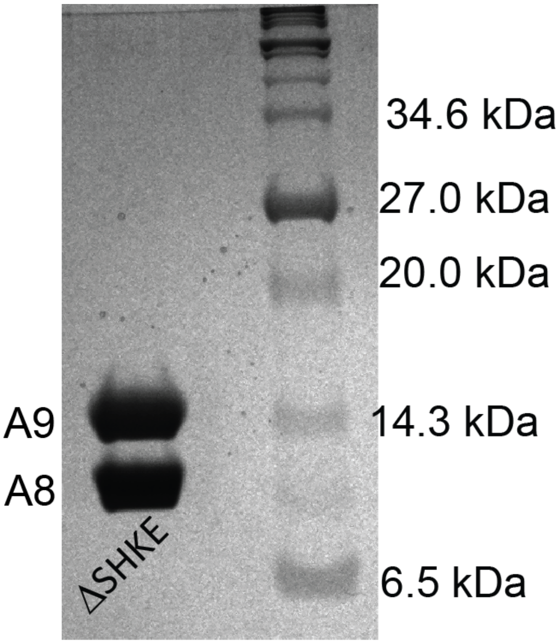

Fig. S2 SDS-PAGE (15% acrylamide Tris-HCl, glycine gels) visualized with Coomassie Blue of purified CP variants used in this study. The ladder is P7702S from New England Biolabs.

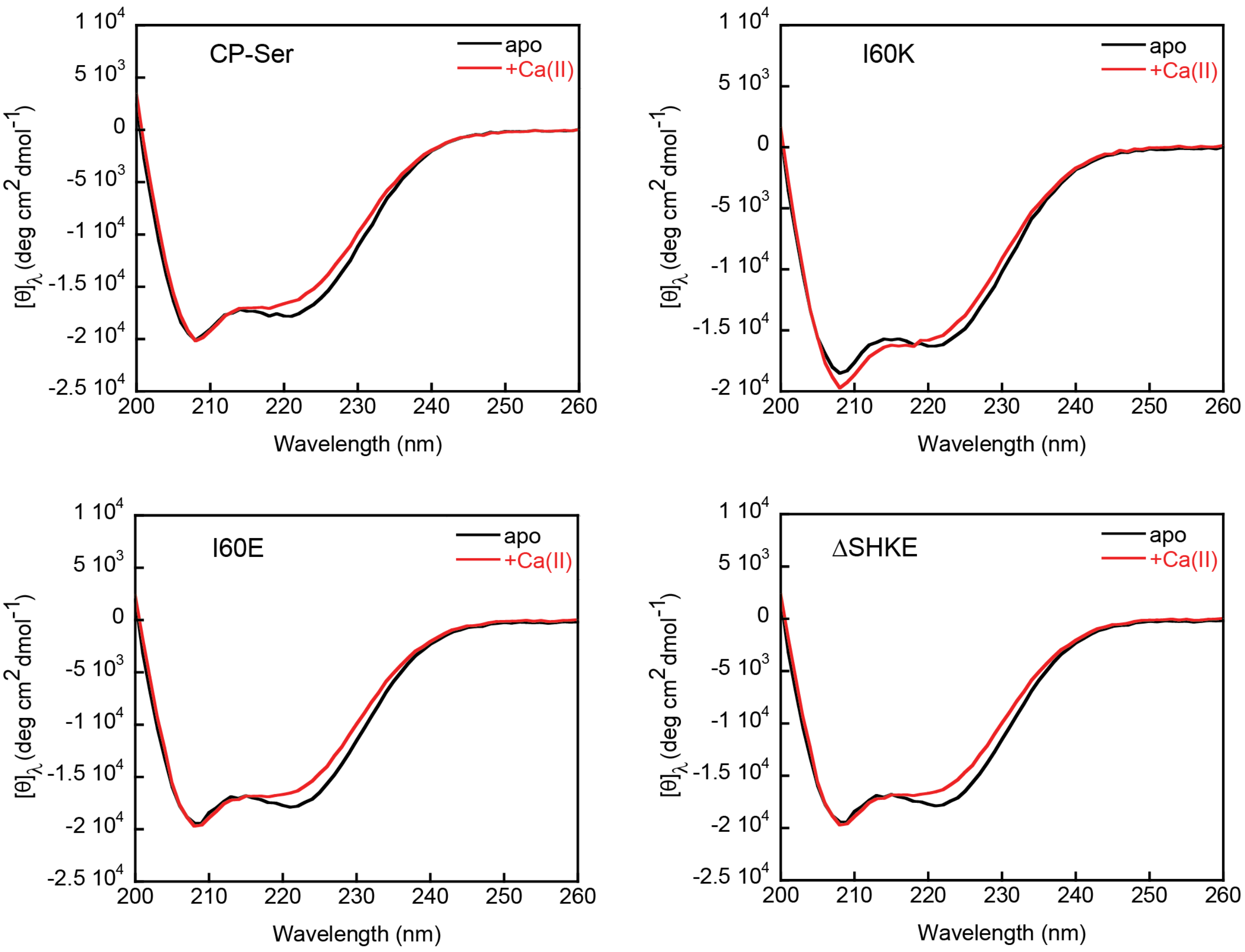

Fig. S3 Circular dichroism spectra of CP-Ser and variants (10 μM) in 1 mM Tris, 0.5 mM EDTA, ±2 mM $CaCl_2$, pH 8.5 at 25 °C.

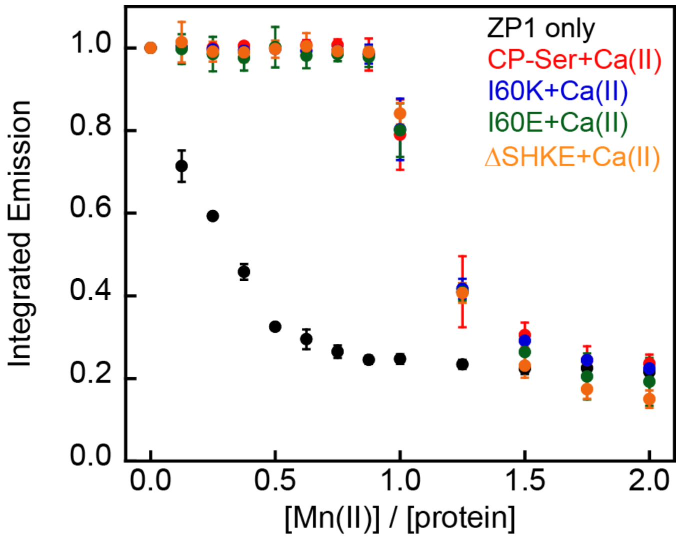

Fig. S4 Competition between ZP1 (1 μM) and CP (4 μM) for Mn(II) in the presence of 200 μM Ca(II) at pH 7.5 (mM HEPES, 100 mM NaCl) and 25 °C (mean ± SDM, *n*=3). Excitation was provided at 490 nm, and the emission spectra were integrated from 500 to 650 nm and normalized with respect to apo ZP1 emission.

ZP1 is a fluorescent small molecule that exhibits fluorescence quenching as a result of Mn(II) binding.[S4] Details of the competition experiment are reported elsewhere.[S5] In this experiment, I60K and I60E behave like CP-Ser and out-compete ZP1 for Mn(II), which indicates high-affinity Mn(II) binding in the presence of excess Ca(II). These data do not provide information about the relative apparent K_d values for Mn(II) for CP-Ser, I60E, and I60K. On the basis of published studies, the K_d value for Mn(II) binding at the His_6 site in the presence of excess Ca(II) is <10 nM.[S5-S7] The apparent $K_{d1,Mn(II)}$ for ZP1 is 550 nM.[S4]

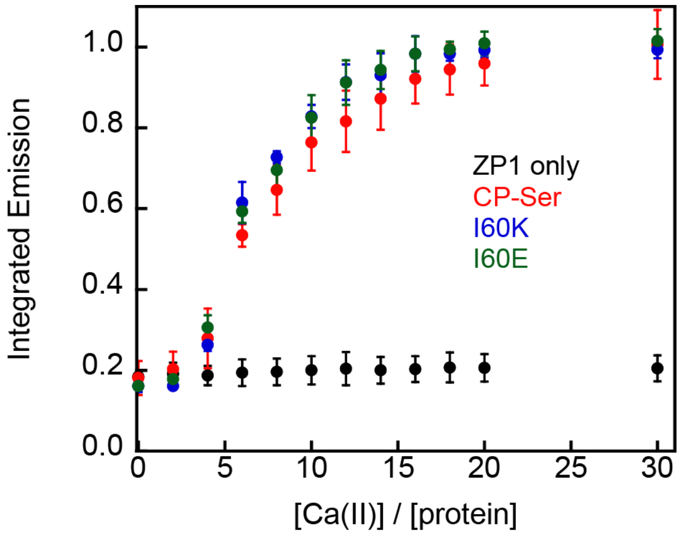

Fig. S5 Competition between ZP1 (1 μM) and CP (4 μM) for 3.5 μM Mn(II) in the presence of increasing concentrations of Ca(II) at pH 7.5 (mM HEPES, 100 mM NaCl) and 25 ^{o}C (mean ± SDM, *n*=3). Excitation was provided at 490 nm, and the emission spectra were integrated from 500 to 650 nm and normalized with respect to apo ZP1 emission.

This experiment shows that CP-Ser, I60K, and I60E require the same number of Ca(II) equivalents to fully activate high-affinity Mn(II) binding and sequester Mn(II) from ZP1. It does not report on the Ca(II) affinities.

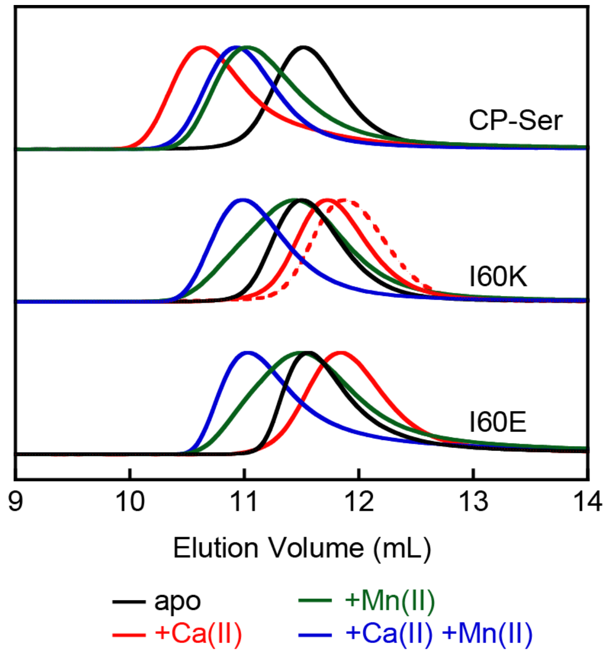

Fig. S6 Size-exclusion chromatography of CP-Ser, I60K, and I60E (30 μM) in the presence of Mn(II) and Ca(II). Black traces, no metal added. Red traces, 600 μM Ca(II) included in the sample and running buffer. Blue traces, 300 μM Mn(II) included in the sample only. Green traces, 600 μM Ca(II) included in the sample and running buffer, 33 μM Mn(II) included in the sample only. The black, red and blue traces correspond to the data in **Fig. 2A** of the main text. All chromatograms were normalized to a maximum absorption of 1. Experiments were performed in 75 mM HEPES, 100 mM NaCl, pH 7.5 at 4 °C. The dashed trace represents data from an experiment performed with 500 μM I60K in 75 mM HEPES, 100 mM NaCl, pH 7.5, 10 mM $CaCl_2$.

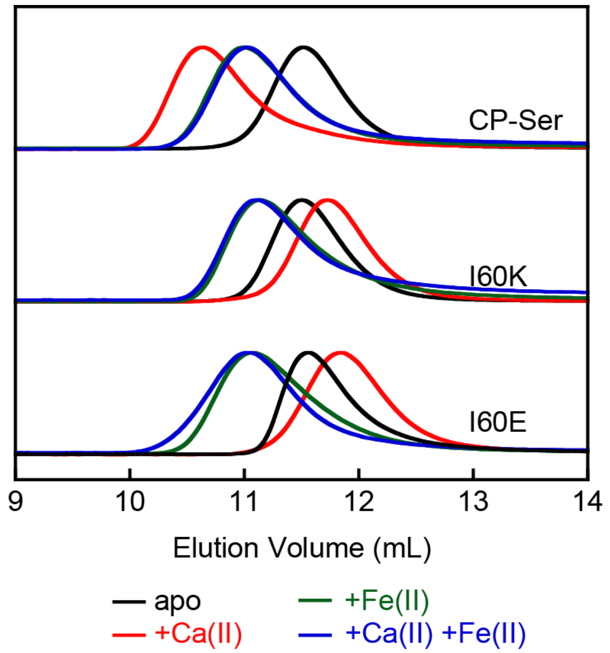

Fig. S7 Size-exclusion chromatography of CP-Ser, I60K, and I60E (30 μM) in the presence of Fe(II) and Ca(II). Black traces, no metal added. Red traces, 600 μM Ca(II) included in the sample and running buffer. Blue traces, 300 μM Fe(II) included in the sample only. Green traces, 600 μM Ca(II) included in the sample and running buffer, Fe μM Mn(II) included in the sample only. The black, red, and blue traces correspond to the data in **Fig. 2B** of the main text. All chromatograms were normalized to a maximum absorption of 1. Experiments were performed in 75 mM HEPES, 100 mM NaCl, pH 7.5 at 4 ^{o}C.

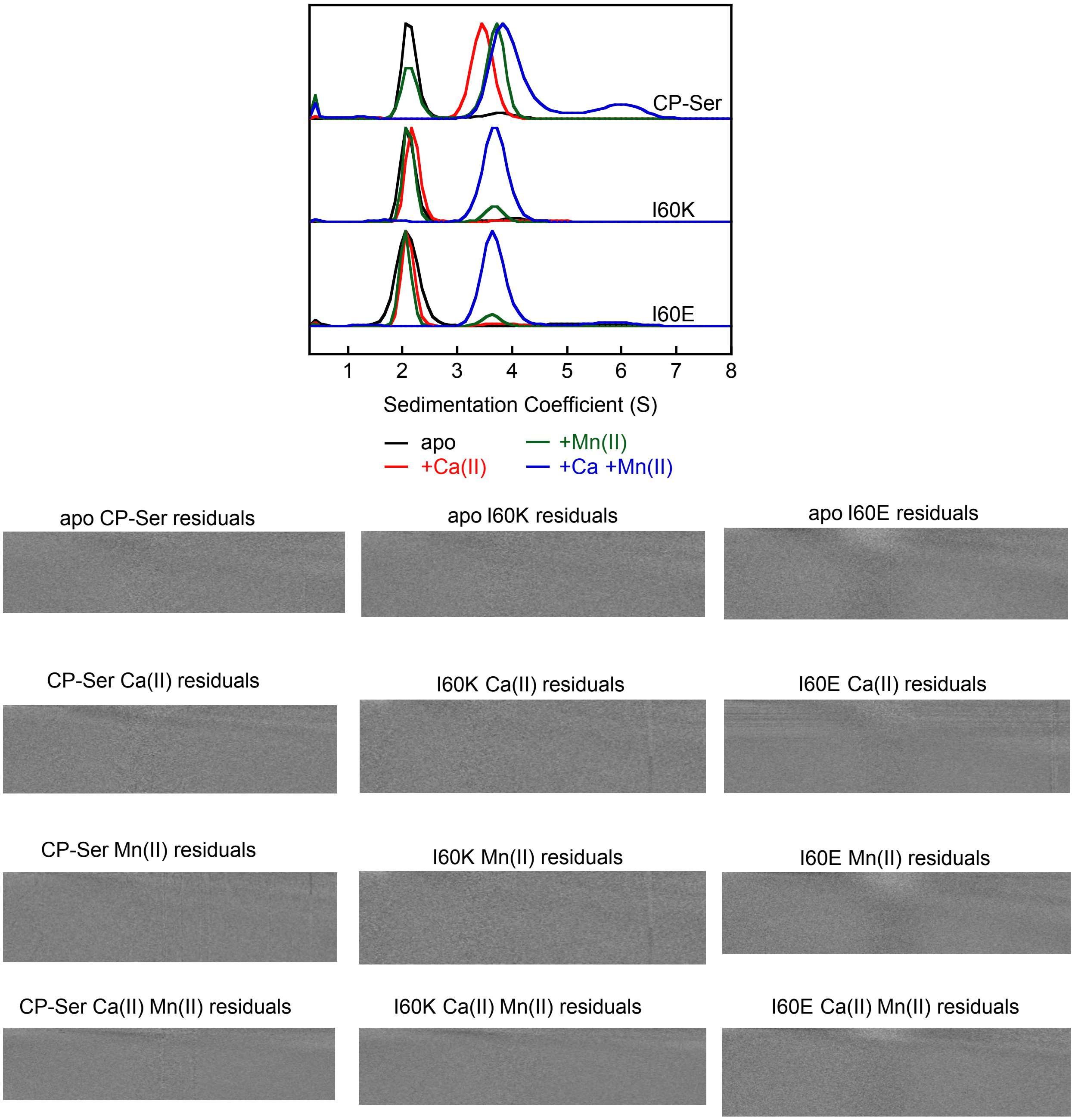

Fig. S8 Normalized sedimentation coefficient distributions of CP-Ser, I60K, and I60E (27.5 μM) and corresponding residuals bitmaps obtained with the *c*(s) model in SEDFIT. Buffer: 75 mM HEPES, 100 mM NaCl, ±540 μM $CaCl_2$, ±27.5 μM $MnCl_2$, pH 7.5 at 20 °C. For apo CP-Ser, 1.35 mM EDTA was included and no metals were added. The data are normalized to a maximum peak height of 1. Each row of pixels in the bitmaps represents an AUC scan with the pixels representing the residuals between the scan and the fit. Darker pixels indicate greater residuals. Diagonal stripes in the bitmaps indicate systematic errors in the fitting. For more information visit: http://www.analyticalultracentrifugation.com/sedfit_help_residuals_bitmap.htm

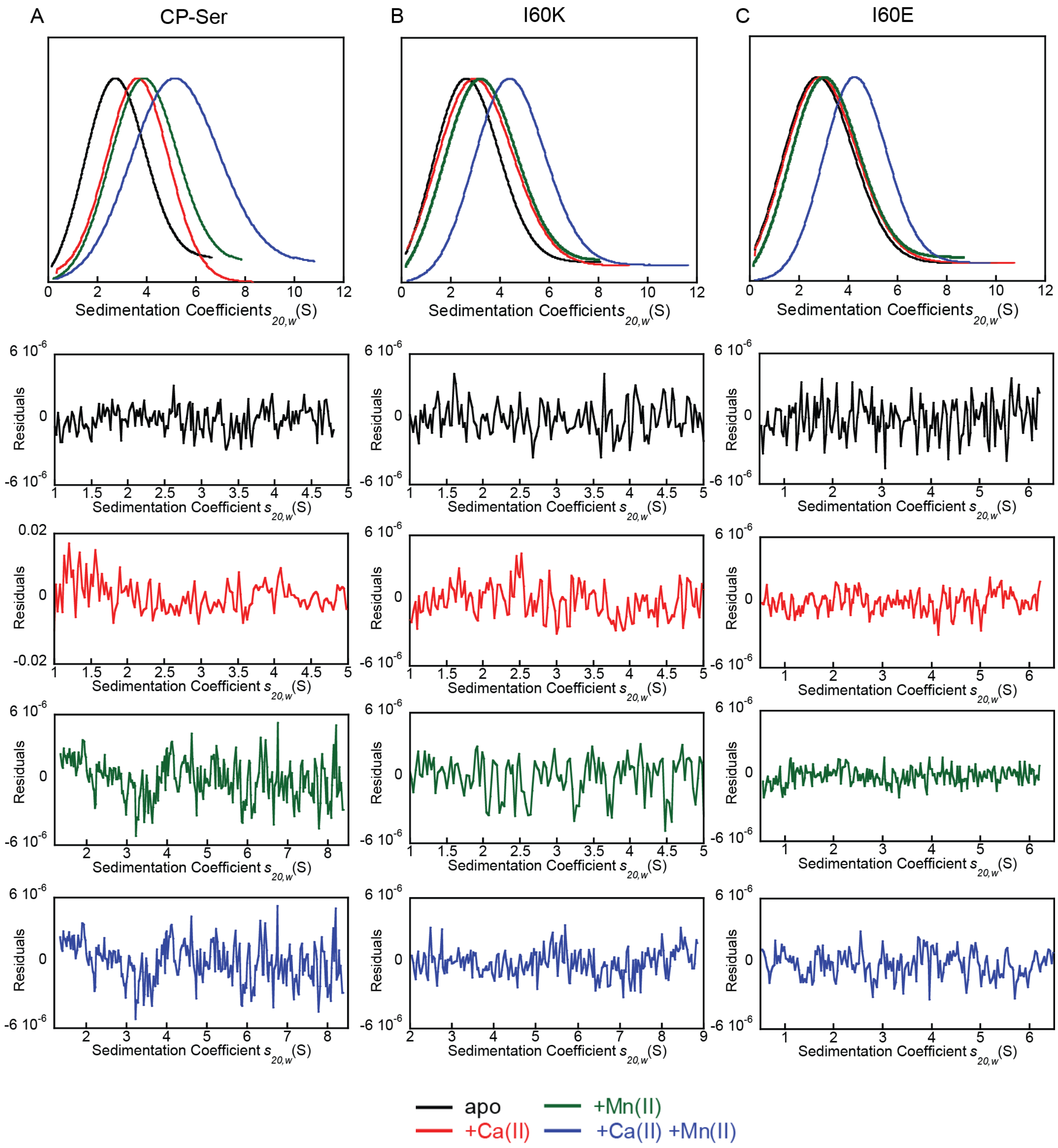

Fig. S9 Sedimentation coefficient distributions and corresponding residuals plots of CP-Ser (A), I60K (B), and I60E (C) (27.5 μM) obtained using DCDT+. Buffer: 75 mM HEPES, 100 mM NaCl, ±540 μM $CaCl_2$, ±27.5 μM $MnCl_2$, pH 7.5 at 20 °C. For apo CP-Ser, 1.35 mM EDTA was included and no metals were added. The data are normalized to a maximum peak height of 1.

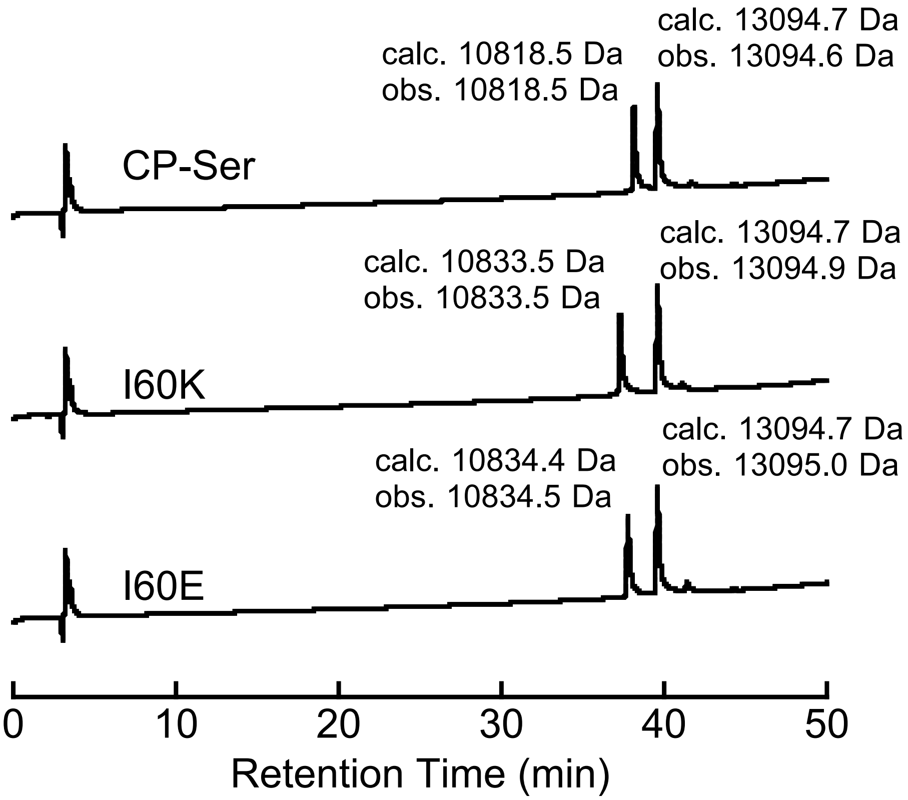

Fig. S10 HPLC chromatograms of CP-Ser, I60E, and I60K (10–60% B over 50 min, 1 mL/min) with corresponding MS data. The peaks for the S100A8 subunits have retention times of ≈38 min and the S100A9 subunit peak occurs at 39.8 min.

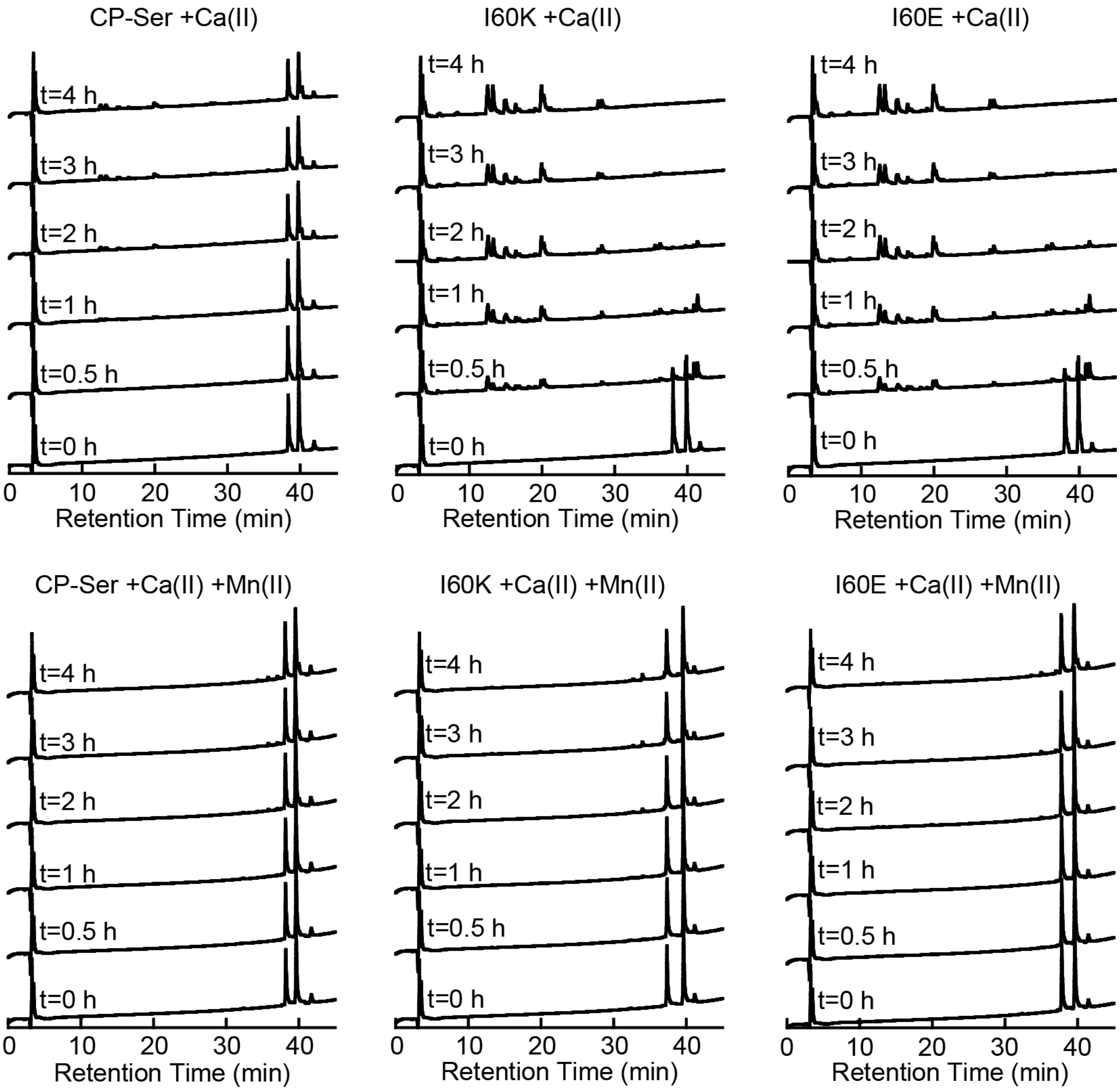

Fig. S11 Full HPLC chromatograms of trypsin (0.45 μM) digestions of CP-Ser, I60E, and I60K (30 μM) in 75 mM HEPES 100 mM NaCl, 1.5 mM $CaCl_2$, ± 30 μM $MnCl_2$, pH 7.5, performed at 37 ^{o}C. These chromatograms correspond to the data presented in **Fig. 4** of the main text.

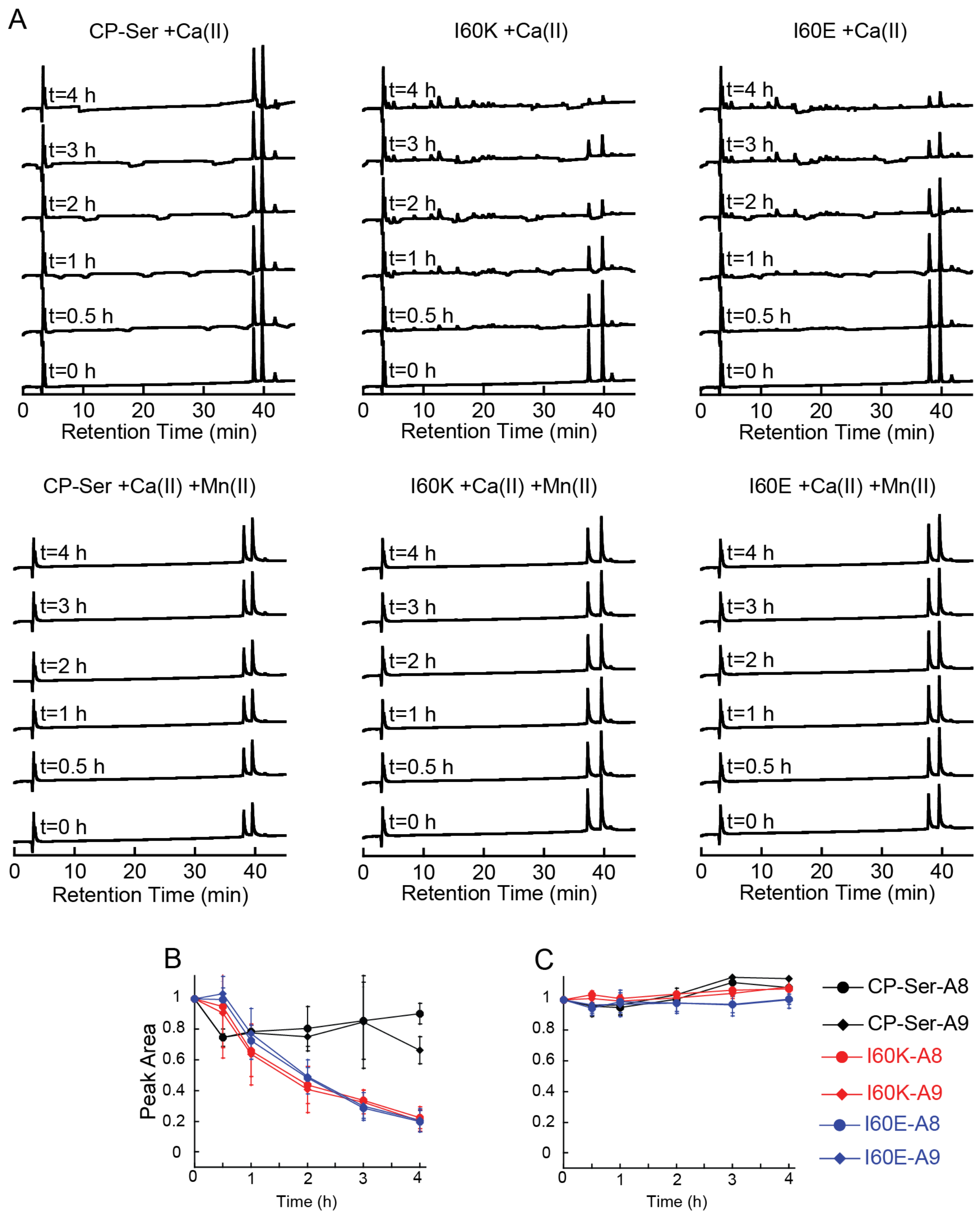

Fig. S12 Susceptibility of Ca(II)-bound CP-Ser, I60E and I60K to degradation by chymotrypsin (0.3 μM). (A) Representative full HPLC traces illustrating the S100A8 and S100A9 subunits following incubation with chymotrypsin for 0–4 h at 37 °C . Digestions were carried out in 75 mM HEPES, 100 mM NaCl, pH 7.5, 1.5 mM $CaCl_2$, ±30 μM $MnCl_2$. (B) S100A8 and S100A9 integrated peak areas as a function of time in the presence of Ca(II) (mean ±SDM, *n*=3). (C) S100A8 and S100A9 integrated peak areas as a function of time in the presence of Ca(II) and Mn(II) (mean ±SDM, *n*=3). The area for each t=0 peak was normalized to 1.

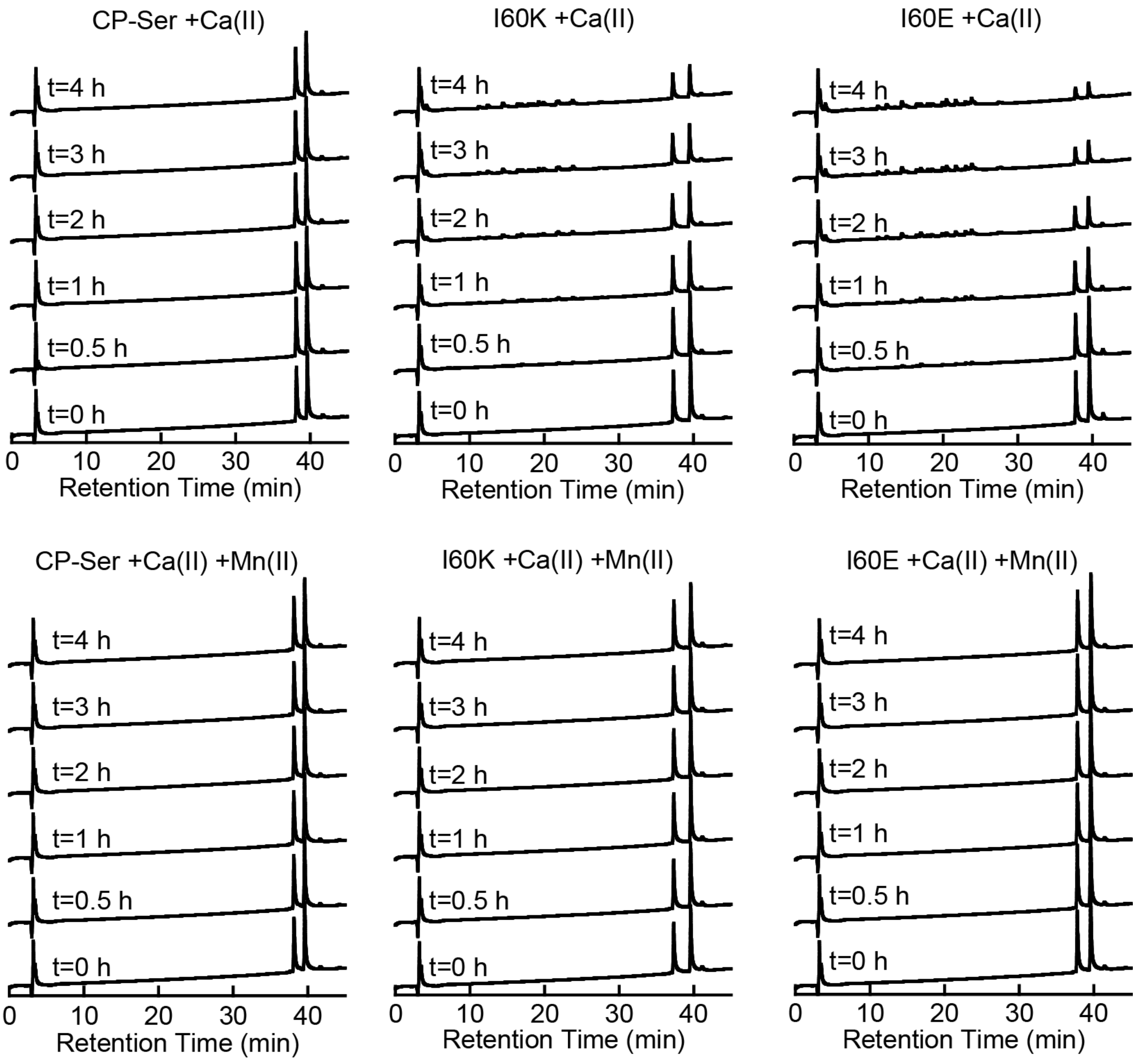

Fig. S13 Full HPLC chromatograms of human neutrophil elastase (0.3 μM) digestions of CP-Ser, I60K, I60E (30 μM) in 75 mM HEPES 100 mM NaCl, 1.5 mM $CaCl_2$, ± 30 μM $MnCl_2$, pH 7.5 performed at 37 °C. These chromatograms correspond to the data presented in **Fig. 5** of the main text.

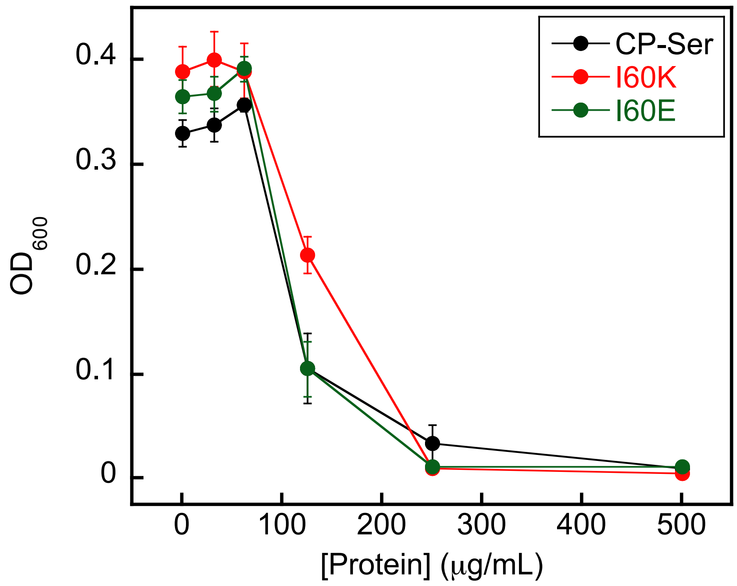

Fig. S14 Antibacterial activity of CP-Ser, I60K, and I60E against *E. coli* ATCC 25922 in the presence of ≈2 mM $CaCl_2$ in the AMA medium. The OD_{600} values were recorded at t = 20 h. Mean ±SEM for three independent replicates (*n*=9).

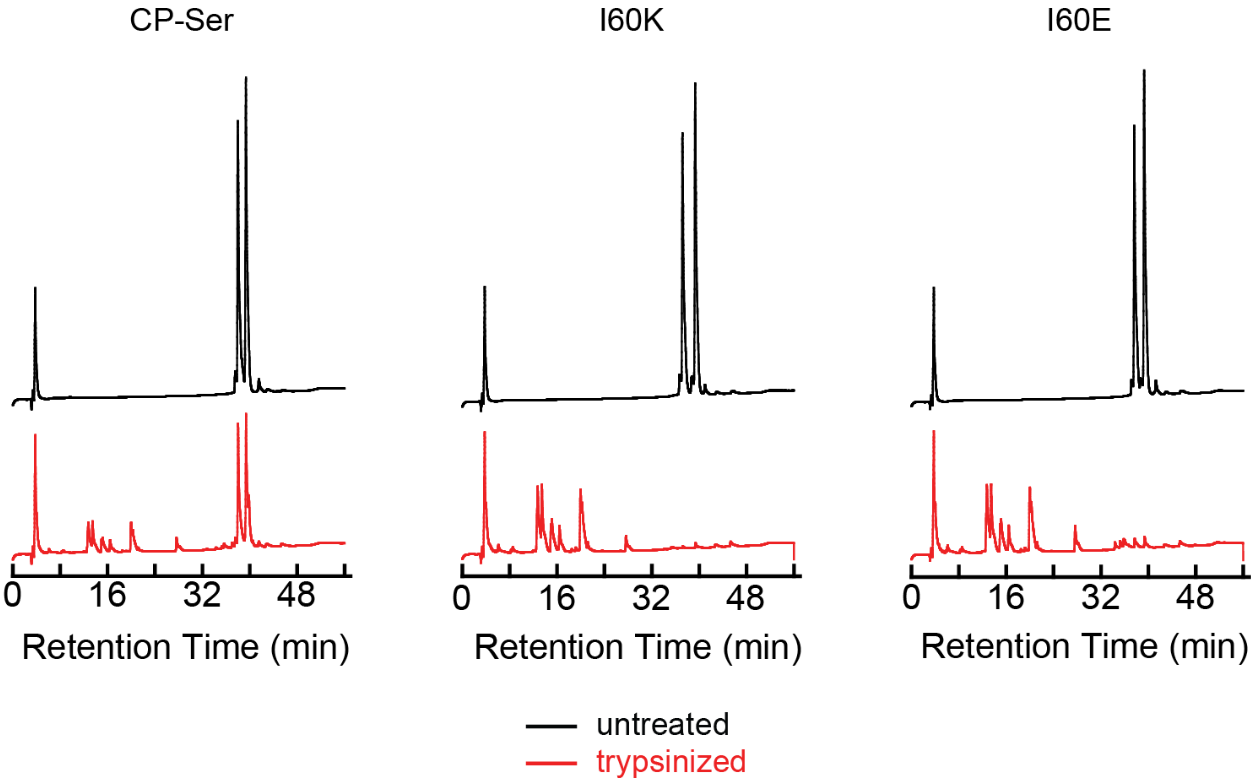

Fig. S15 HPLC chromatograms (10–60% B over 50 min, 1 mL/min) of proteins employed in the antibacterial activity assays evaluating the effect of pre-incubation with trypsin. The proteins (210 μM) were incubated at 37 °C in 20 mM Tris, 100 mM NaCl, 3 mM $CaCl_2$, pH 7.5, ±0.45 μM trypsin for ≈20 h prior to the antimicrobial activity assays. The HPLC traces were acquired following the ≈20 h incubation. The data for the antimicrobial activity assays are presented in **Fig. 6** of the main text.

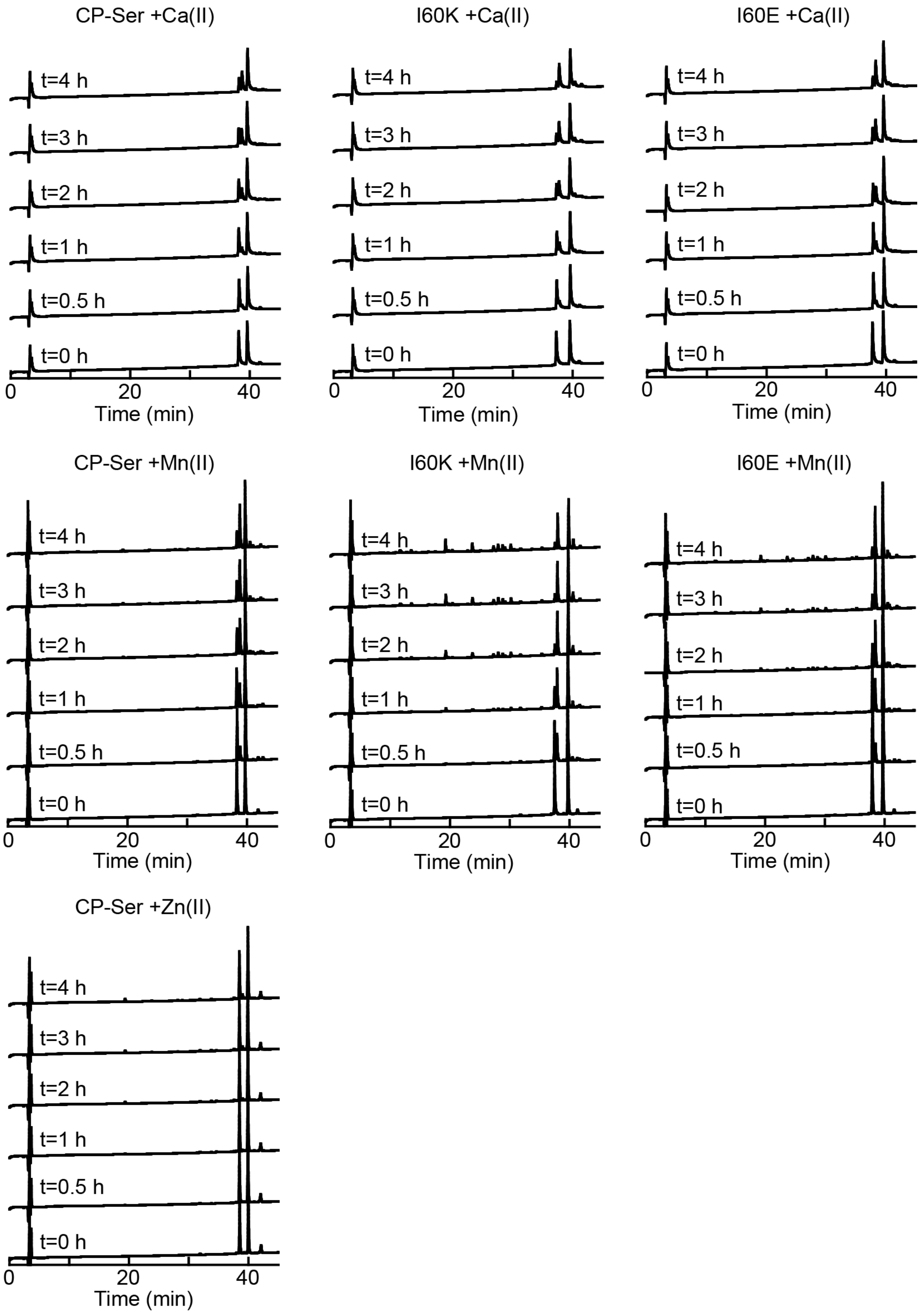

Fig. S16 Full HPLC chromatograms of GluC (0.3 μM) digestions of CP-Ser, I60K, I60E (30 μM) in 75 mM HEPES 100 mM NaCl, ±1.5 mM $CaCl_2$, ±30 μM $MnCl_2$, pH 7.5. A digestion of CP-Ser with 60 μM $ZnCl_2$ is also shown. All experiments were performed at 37 °C. These chromatograms correspond to the data presented in **Fig. 7** of the main text.

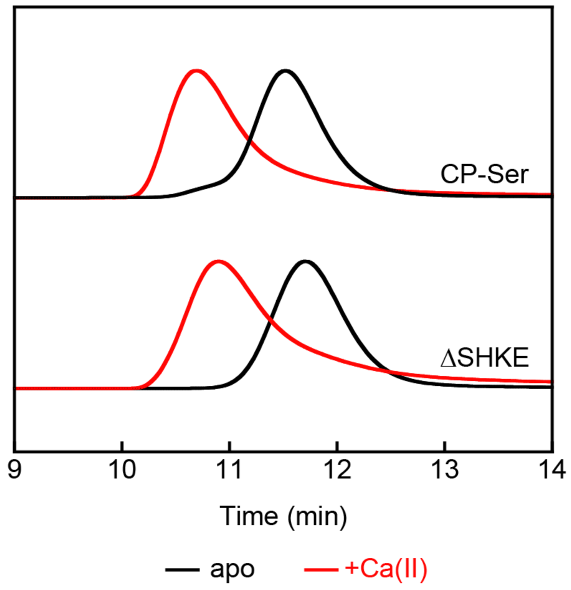

Fig. S17 Size-exclusion chromatography of CP-Ser and ΔSHKE (30 μM) performed with no metal added (black trace) and 600 μM Ca(II) included in the sample and running buffer (red trace). The chromatograms were normalized to a maximum absorption of 1. Experiments were performed in 75 mM HEPES, 100 mM NaCl, pH 7.5 at 4 °C.

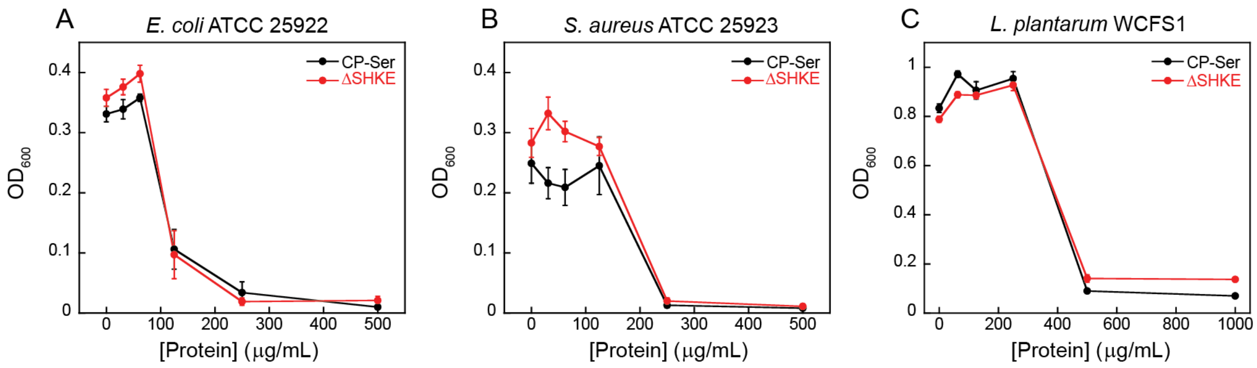

Fig. S18 Antibacterial activity of CP-Ser and ΔSHKE against *E. coli* ATCC 25922 (A), *S. aureus* ATCC 25923 (B), *L. plantarum* WCSF1 (C) with 2 mM $CaCl_2$ in the AMA medium. The OD_{600} values were recorded at t = 20 h. Averages ±SEM for three independent replicates (*n*=9).

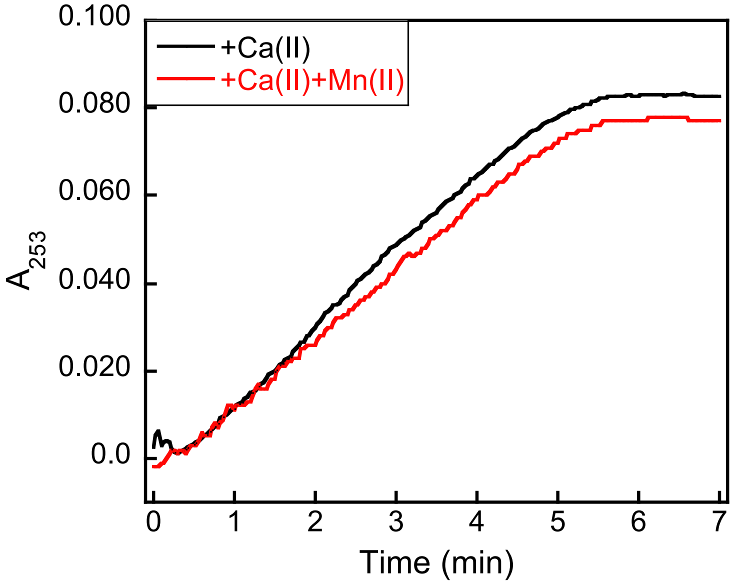

Fig. S19 Effect of Mn(II) on trypsin activity. Trypsin activity was monitored by N^{α}-benzoyl-L-arginine ethyl ester cleavage in 75 mM HEPES, 100 mM NaCl, pH 7.5, 1.5 mM Ca(II), ±30 μM $MnCl_2$. Averages of three trials are shown.

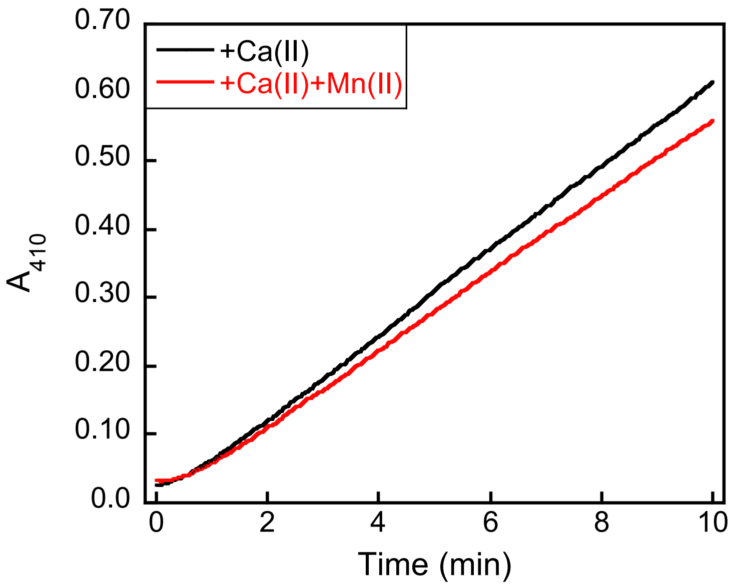

Fig. S20 Effect of Mn(II) on chymotrypsin activity. Activity of chymotrypsin was monitored by cleavage of *N*-Succinyl-Ala-Ala-Pro-Phe *p*-nitroanilide in 75 mM HEPES, 100 mM NaCl, pH 7.5, 1% DMF, 1.5 mM $CaCl_2$, ±30 μM $MnCl_2$ at 25 °C. Averages of three experiments are shown.

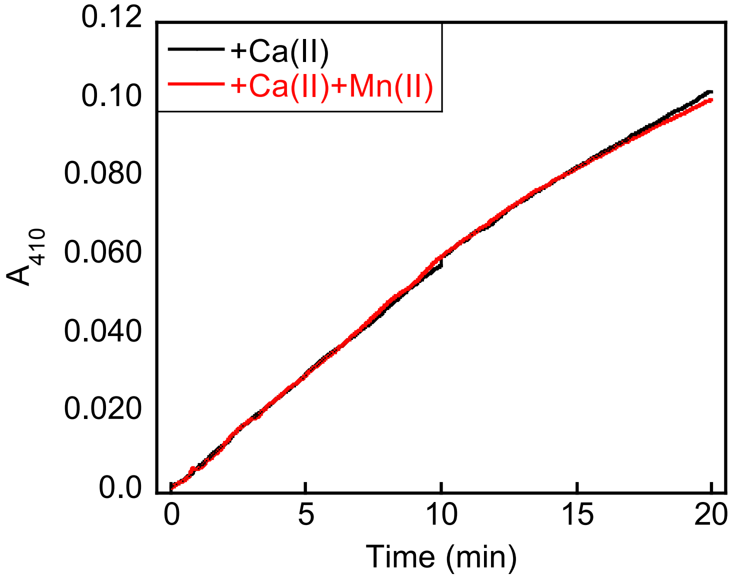

Fig. S21 Effect of Mn(II) on human neutrophil elastase activity. Activity of human neutrophil elastase was monitored by cleavage of *N*-Succinyl-Ala-Ala-Val-Ala *p*-nitroanilide in 75 mM HEPES, 100 mM NaCl, pH 7.5, 5% DMSO, 1.5 mM $CaCl_2$, ±30 μM $MnCl_2$ at 25 ^{o}C. Averages of three experiments are shown.

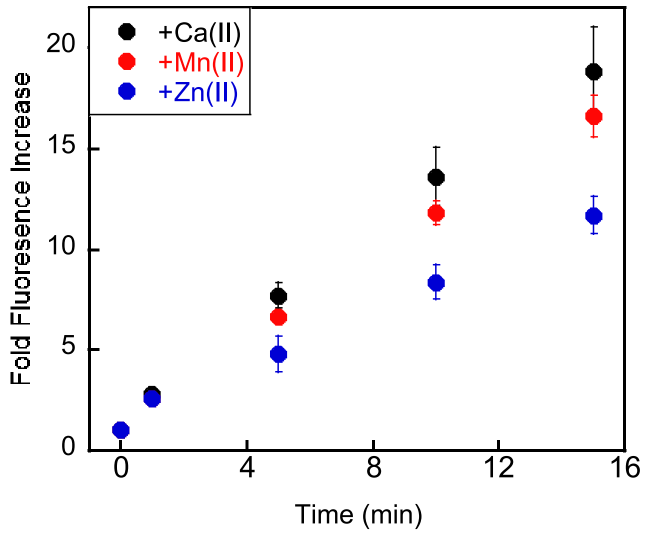

Fig. S22 Effect of Mn(II) and Zn(II) on GluC activity. Activity of GluC as monitored by cleavage of carboxybenzyl-Leu-Leu-Glu-β-napthylamide in 75 mM HEPES, 100 mM NaCl, 10% DMSO, pH 7.5 with 1.5 mM $CaCl_2$, 30 μM $MnCl_2$, 60 μM $ZnCl_2$ (average ±SDM, *n*=3). The zinc conditions also contained 30 μM CP-Ser because precipitation occurred when Zn(II) was added to solutions of substrate.

Supplementary References

(S1) M. B. Brophy, J. A. Hayden, E. M. Nolan, *J. Am. Chem. Soc.* 2012, **134**, 18089-18100.

(S2) E. Shaw, M. Mares-Guia and W. Cohen, *Biochemistry*, 1965, **4**, 2219-2224.

(S3) M. M. Santoro and D. W. Bolen, *Biochemistry*, 1988, **27**, 8063-8068.

(S4) Y. You, E. Tomat, K. Hwang, T. Atanasijevic, W. Nam, A. P. Jasanoff and S. J. Lippard, *Chem. Commun.* 2010, **46**, 4139-4141.

(S5) J. A. Hayden, M. B. Brophy, L. S. Cunden and E. M. Nolan, *J. Am. Chem. Soc.* 2013, **135**, 17804-17817

(S6) T. E. Kehl-Fie, S. Chitayat, M. I. Hood, S. Damo, N. Restrepo, C. Garcia, K. A. Munro, W. J. Chazin, and E.P. Skaar, *Cell Host Microbe*, 2011, **10**, 158-164.

(S7) M. B. Brophy and E. M. Nolan, *ACS Chem. Biol.*, 2015, **10**, 641-651.
